# The cost of health workforce gaps and inequitable distribution in the Ghana Health Service: an analysis towards evidence-based health workforce planning and management

**DOI:** 10.1186/s12960-021-00590-3

**Published:** 2021-03-31

**Authors:** James Avoka Asamani, Hamza Ismaila, Anna Plange, Victor Francis Ekey, Abdul-Majeed Ahmed, Margaret Chebere, John Koku Awoonor-Williams, Juliet Nabyonga-Orem

**Affiliations:** 1 Ghana Health Service, Private Mail Bag, Accra, Ghana; 2 Universal Health Coverage - Life Course, Inter-Country Support Team for Eastern and Southern Africa, World Health Organization, Regional Office for Africa, Harare, Zimbabwe

**Keywords:** Human Resources for Health, Maldistribution, Health Workforce Distribution, Staffing norms, Human Resource Gaps Analysis, Health Workforce policy

## Abstract

**Background:**

Despite tremendous health workforce efforts which have resulted in increases in the density of physicians, nurses and midwives from 1.07 per 1000 population in 2005 to 2.65 per 1000 population in 2017, Ghana continues to face shortages of health workforce alongside inefficient distribution. The Ministry of Health and its agencies in Ghana used the Workload Indicators of Staffing Needs (WISN) approach to develop staffing norms and standards for all health facilities, which is being used as an operational planning tool for equitable health workforce distribution. Using the nationally agreed staffing norms and standards, the aim of this paper is to quantify the inequitable distribution of health workforce and the associated cost implications. It also reports on how the findings are being used to shape health workforce policy, planning and management.

**Methods:**

We conducted a health workforce gap analysis for all health facilities of the Ghana Health Service in 2018 in which we compared a nationally agreed evidence-based staffing standard with the prevailing staffing situation to identify need-based gaps and inequitable distribution. The cost of the prevailing staffing levels was also compared with the stipulated standard, and the staffing cost related to inequitable distribution was estimated.

**Results:**

It was found that the Ghana Health Service needed 105,440 health workers to meet its minimum staffing requirements as at May 2018 vis-à-vis its prevailing staff at post of 61,756 thereby leaving unfilled vacancies of 47,758 (a vacancy rate of 41%) albeit significant variations across geographical regions, levels of service and occupational groups. Of note, the crude equity index showed that in aggregate, the best-staffed region was 2.17 times better off than the worst-staffed region. The estimated cost (comprising basic salaries, market premium and other allowances paid from central government) of meeting the minimum staffing requirements was estimated to be GH¢2,358,346,472 (US$521,758,069) while the current cost of staff at post was GH¢1,424,331,400 (US$315,117,566.37), resulting in a net budgetary deficit of 57% (~ US$295.4 million) to meet the minimum requirement of staffing for primary and secondary health services. Whilst the prevailing staffing expenditure was generally below the required levels, an average of 28% (range 14–50%) across the levels of primary and secondary healthcare was spent on staff deemed to have been inequitably distributed, thus providing scope for rationalisation. We estimate that the net budgetary deficit of meeting the minimum staffing requirement could be drastically reduced by some 30% just by redistributing the inequitably distributed staff.

**Policy implications:**

Efficiency gains could be made by redistributing the 14,142 staff deemed to be inequitably distributed, thereby narrowing the existing staffing gaps by 30% to 33,616, which could, in turn, be filled by leveraging synergistic strategy of task-sharing and/or new recruitments. The results of the analysis provided insights that have shaped and continue to influence important policy decisions in health workforce planning and management in the Ghana Health Service.

**Supplementary Information:**

The online version contains supplementary material available at 10.1186/s12960-021-00590-3.

## Introduction

Globally, many health systems continue to grapple with a myriad of human resources for health (HRH) challenges such as mismatches between the need for, demand for and supply of health workforce which are largely underpinned by low levels of training outputs, insufficient remuneration, inadequate funding and migration among others [1]. Particularly in the Africa Region, health workforce-related challenges remain one of the major threats to the attainment of the Sustainable Development Goal (SDG) three, including Universal Health Coverage (UHC). If the lessons from the erstwhile Millennium Development Goals (MDGs) are anything to go by, the degree of success in this respect will be highly linked to the availability, accessibility and quality of the health workforce, an element of the health system that remains thorny; and has been linked to observed disparities in some health outcomes [2, 3]. There are, therefore, genuine concerns about the ability of the countries to step up efforts for the realisation of the SDGs, particularly SDG3 given the chronic health workforce challenges confronting the Africa region.

In the context of Ghana, various initiatives and activities are being implemented or scaled-up towards the attainment of UHC and SDG 3 by the year 2030 [4]. The Ghana National Health policy, the Health Sector Medium Term Development Plan (HSMTDP 2018–2021 and earlier ones) are all geared towards ensuring Universal Health Coverage and strengthening the health system to effectively respond to the health needs of its citizens including health emergencies [5–8]. Consequently, there have been enormous efforts in the expansion of healthcare infrastructure, social health insurance coverage, as well as training and employment of the health workforce.

Ghana has over the last decade increased the production and retention of its health workforce resulting in tremendous increases in the density of physicians, nurses and midwives from 1.07 per 1000 population in 2005 to 2.65 per 1000 population in 2017 [9]. These efforts have not only resulted in Ghana being cited as a country on a good footing towards UHC but also as a leading producer of physicians, nurses and midwives in sub-Saharan Africa [10]. Nonetheless, some reports and published literature assert that Ghana’s HRH stock may not be optimal and is plagued with inefficient distribution [11–14]. For instance, Scheffler et al. [14] showed a serious deficit in the number of physicians, nurses and midwives in Ghana by 2015 which later estimates puts at 42% gross deficit in HRH availability but much worse amongst specialised groups of health professionals [15].

The maldistribution of available staff has manifested either as aggregate (absolute) or relative (skill-mix distortions) [16]. Aggregate or absolute maldistribution occurs when the composite of the HRH is distributed in a manner skewed against geographical region(s) or special population groups. On the one hand, relative maldistribution or skill-mix distortion is said to have occurred when highly skilled health workers are concentrated in certain locations (usually urban areas), leaving other locations (usually rural and under-served areas) with low skilled workers [17]. In either situation, the population is likely to seek health services from the health facilities in locations with the requisite HRH which in turn increases workload in those facilities giving rise to a legitimate clamour for more staff. If not addressed, this forms a vicious cycle of ‘inequity breeding inequity’. A redistribution of the health workforce based on needs assessment using a nationally agreed evidence-informed standard or norm can be an important step to addressing the inequalities in HRH distribution and its impact on health care delivery.

As part of efforts to address HRH shortages and maldistribution within the health sector of Ghana, the Ministry of Health (MOH) and its partners developed a staffing standard (known as staffing norms) for publicly funded healthcare facilities in the country [18]. This HRH planning tool, developed based on the World Health Organization (WHO) recommended Workload Indicators of Staffing Needs (WISN) method, gives an indication of the calibre and number of health workers required in a given health care setting based on their workload. The process of WISN for development of staffing is reported elsewhere [19].

Notwithstanding that the staffing norms have been widely accepted by stakeholders and are being used by the Ghana Health Service (GHS) since 2015 for workforce planning and deployment, turning the tide of inequitable health workforce distribution remains a challenge. This is also in a context where several studies have revealed significant health system inefficiencies, including the health workforce [20–23]. For instance, it is estimated that each health centre in Ghana could save at least US$7,062 annually if they were more efficient [22] which represents about 15% of their US$44,638 annual budgetary requirement for service delivery [24]. A large portion of the inefficiencies has been attributed to the health workforce in terms of inefficient distribution [25] and sub-optimal productivity [26].

To address the aforesaid challenge from a health workforce planning perspective, Ministry of Health (MOH) and its partners have been desirous of an analysis of the workforce gaps and cost based on the newly developed staffing norms [27]. This paper seeks to illustrate the use of a nationally agreed health facilities staffing standard to identify in aggregate terms, the inequitable distribution of the health workforce and associated cost implications as well as how it is being used to shape health workforce policy, planning and management.

### Overview of the public health sector in Ghana

Ghana’s population was estimated at 28,687,274 (the year 2016) with an annual growth rate of 2.7% [28]. The country at the time of analysis was divided into ten (10) political and administrative regions, which were further divided into 254 districts (the country’s regions increased to 16 and the districts increased to 260 in 2019). Each district is also divided into sub-districts and communities which health service delivery is administratively and operationally aligned with. The GHS and Teaching Hospitals Act, 1996, Act 525 vests in the GHS the mandate to provide primary and secondary health care services to all people living in Ghana. This is complemented by private institutions and quasi-governmental institutions. The Teaching Hospitals, as per Act 525, are mandated with the provision of tertiary health care services to the people living in Ghana. This leaves the Ministry of Health with a core responsibility for policy formulation and resource mobilisation.

In descending order of complexity in the health service delivery hierarchy are Teaching Hospitals (THs) at the top, which are semi-autonomous national referral hospitals with a mandate of managing complex health problems, research and staff training. Each TH is linked to a university to enhance its functions. The GHS, which is by far the largest health service delivery agency of the MOH, provides about 60% of outpatient and inpatient services and nearly all preventive public health care services [6]. In total, the GHS managed about 4507 health facilities in 2018, of which 0.2% were Regional Hospitals or secondary level facilities. The rest were primary level facilities comprising 3% District (Primary) Hospitals, 19.3% Health Centres, 0.8% Polyclinics and 77% Community-based Health Planning and Service (CHPS) [29], deemed the vehicle for delivering Primary Health Care (PHC) [4]. See Table 1 for the number of health facilities managed by GHS.

**Table 1:** Health facilities managed by GHS, 2018

Type of health facility	Number	Percentage (%)
CHPS	3,463	76.8
Health Centre	871	19.3
Polyclinic	36	0.8
District (Primary) Hospital	127	2.8
Regional Hospital	10	0.2
Grand total	4507	

Source: District Health Management Information System-2 (DHIMS-2)

The Regional Hospitals (RHs) are situated in the regional capitals to provide secondary level of specialised health care and serve as referral centres for all District Hospitals in each region. On the other hand, District Hospitals (DHs) provide basic and emergency healthcare and have catchment areas coterminous with political districts or a population of 100,000–200,000. Sub-districts are served by Health Centres (HCs), which provide basic curative and preventive services. These are intended to serve populations of about 20,000. In urban areas, however, their capacity may be enhanced to become polyclinics where they serve populations larger than 20,000. At the community level, the main service delivery facilities for preventive services and treatment of minor ailments are CHPS that may have a physical structure (Compound) or not. These are intended to serve a population of 5000 or 750 households and maybe coterminous with electoral areas [4]. The statistics reported in this paper relate to health facilities managed by GHS, from Regional Hospitals through District Hospitals, Health Centre/Polyclinics to CHPS (Table 1). Thus, Teaching Hospitals, Mission Health Facilities, Quasi-Government Health Facilities and Private-for-Profit Health Facilities have not been included in the analysis due to data-related challenges.

## Tools and methods

### An overview of the WISN implementation and staffing norms development in Ghana

The MOH and its agencies, notably the Ghana Health Service, adopted the workload indicators of staffing needs (WISN) methodology in 2011 for health workforce planning in the country. Extensive country-wide data collection and analysis using this tool resulted in the development of staffing norms and standards for all health workers and health facilities in Ghana, which was completely adopted by government as a national health sector staffing policy in 2018. The staffing norms and standards provide benchmarks in terms of overall patient volumes for health facility types and the corresponding staffing numbers required to cope with the workload. The methodology of WISN application in Ghana and the development of the staffing norms have been documented elsewhere [19]. Table 2 provides a summary of the essential steps that were taken in the country-wide application of WISN and how the WISN results were used to develop the staffing norms. With permission from the GHS, relevant sections of the staffing norms and standards are included as Additional file 1 in which the staffing norms and standards by health facility type are contained in Additional file 1: Table S1–9 for ease of reference.

**Table 2:** A summary of the WISN implementation and staffing norms development process in Ghana

No.	Generic WISN steps	How it was applied in Ghana
1	Governance and technical processes	Following a capacity building workshop facilitated by WHO, a National Steering Committee (NSC) was established to provide political and technical leadership for the application of WISN for the purpose of developing staffing norms in Ghana. The NSC also led in mobilising funding for the process. A 17-member Technical Working Group (TWG), drawn from various agencies was also constituted to undertake the WISN application. The TWG routinely reported the progress of work to the NSC and received guidance as and needed. In each health facility that was visited, an Expert Group was formed by occupational category to assist in the setting of activity standards
2	Determining the priorities for WISN application	Based on the policy direction of the Ministry of Health, it was prioritised to apply WISN for all health workers in the country (across 141 categories of clinical and non-clinical staff). In a first phase from 2013 to 2014, 70% of the categories prioritised were covered while the rest were covered in a second phase in 2017–2018
3	Estimating available working time (AWT) for health professionals	In determining the AWT, national leave policy which stipulates the number of days each category of health workers was entitled to was used, alongside, the average number of sick leave taken by health workers which was obtained from each of the health facilities visited, national public holidays, average maternity leave and training days per year were deducted from the total annual working days
4	Defining the workload components	The workload components were defined as the tasks (duties) performed by staff on a typical day. These workload components were classified into three: Health Service Activities (or Administrative Activities in case of administration staff), Support activities and Additional activities *Health Service Activities* refer to tasks performed by all members of a staff category for which regular statistics are collected. Example, number of deliveries, OPD, surgeries etc * Support activities* are tasks performed by *all* members of a staff category, but statistics are *not* collected regularly. Example, documentation of patient care, meetings, etc. * Additional activities* are tasks performed by *some (not all)* members of a staff category, but statistics are *not* collected regularly. Example, administrative duties Data was collected from 54 randomly selected health facilities and institutions to develop the workload components and activity standards which was validated and applied in a nationally representative sample of 138 health facilities countrywide across all levels of the public health system. Expert Groups were formed at the health facilities visited who provided technical insights into their work determine the workload components using a purposely deigned job components tool
5	Setting activity standards	Activity Standard (or Service Standard) is the time it takes a trained and well-motivated member of a particular staff category to perform his/her duties to acceptable professional standards in the circumstances of the country/facility. Setting of the activity standards was undertaken concurrently with that of the workload components. Aimed to achieve a technical consensus, the Expert Group in the first health facility provided a list of health service activities they perform, and the corresponding time spent on each. These were then collected and sent to the next health facility, where the completed tool was given to another batch of health professionals (in the same category) to indicate if they agreed with the previous batch of health professionals' proposal. The process continued until a near consensus was achieved where no new workload components were added and the standard time acceptable to all. Where there were still divergent views, non-obtrusive direct observation to determine the standard time was carried out. In all the 192 health facilities used (54 pilot sites for workload components and activity standards development and 136 scale-up application), the institutional staffing requirement was calculated and discussed with the health workers and their management whose comments were used to refine the analysis
6	Establishing standard workloads	Standard Workload is the amount of work (within one activity) that one person could do in a year. Standard Workload is the Available working time divided by the activity standard (Service Standard) of a particular task *(Standard Workload* = *AWT ÷ Activity Standard)* Standard workload for all activities and categories of staff were determined with the aid of the WISN software
7	Calculating allowance factors	Allowance factor (AF) is the estimation of the number of health workers required to cover support activities and additional activities. There are two types of allowance factors—category and individual The category allowance factor (CAF) is a multiplier that is used to calculate the total number of health workers, required for both health service and support activities The individual allowance factor (IAF) is the staff required to cover additional activities of certain cadre members These calculations have been automated in the WISN software
8	Determination of staff requirements	In determining the staff requirements, in each health facility, the annual workload statistics was obtained from the annual report, health information system and admission and discharge books in the wards, as appropriate. For each workload component, the annual service statistics was used to divide by its respective standard workload. A sum of all workload components was then put together to get the total staff requirement for all health service activities. The allowance factors are then applied to get the true staffing requirement using the formula below *Total required number of staff = (A*×*B) + C*, where A = required staff for health service activities B = category allowance factor C = individual allowance factor The staffing requirements for the individual staff categories and health facilities/institution was computed using the stated formula using the WISN software
9	Development of national staffing norms from the WISN analyses	The facility-level WISN results (staffing requirements) were validated and meta-analysed to establish a national staffing norm for the various categories of health facilities. This process included data preparation and validation, statistical analysis for setting staffing norms and validation *(a)* *Data preparation* facility-based WISN results were compiled in an excel template for inspection and comparison by facility and staff category. Each facility WISN output (staffing requirement) was assessed for internal and external validity. For internal validation, the facility WISN output was checked to see if the results generally made sense in the light of expert knowledge about the general staffing situation in that facility; and for relativities among cadres in the facility—for example the ratio of doctors to nurses from the WISN results. For external validation, each facility WISN output was assessed to find out if there is any significant difference between that facility and others of similar status and service utilisation. Health facilities were then grouped into workload categories. Whenever unexplained discrepancies were detected, a verification of the inputted data vis-à-vis expert consultation and a re-run of the WISN study was made to correct the errors (if any) *(b)* *Determining the national staffing norms from WISN results: The facility based WISN results (grouped by type of health facility and similarity of workload) was* meta-analysed using random effect model of meta-analysis (the random effect model assumes that when pooling results, there could have been variations within and across studies). The pooled average staffing requirements for each cadre based on the meta-analysis and its boundaries of uncertainties (95% confidence limits) were considered the ‘statistical limits’ for setting the staffing norms: The lower limit of the 95% confidence interval of the pooled mean requirement of each category of the staff was considered the minimum staffing limit on the staffing norm for that cadre The upper limit of the 95% confidence interval of the pooled mean requirement of each category of the staff was considered the maximum staffing limit on the staffing norm *(c) Validation and adoption The* draft staffing norm was then reviewed and validated in a series of consultation and validation workshops across the country with stakeholders across all levels of the health system including health professions regulators, labour unions, and frontline health managers. Feedback from the series of validation workshop was used to finalise the staffing norms document before it was adopted as a national policy for health workforce planning, distribution and management in the public health sector

### Analytical framework

Based on the health sector staffing norms and standards [19, 30], the prevailing staffing situation of all health facilities of GHS was compared with their staffing requirement in which two levels of comparison were made: absolute HRH gaps and relative HRH gaps (Staff Availability Ratio).

### Establishing absolute HRH gaps

The difference between the current staffing levels and the required number for a particular cadre in a facility was considered as the Absolute HRH Gap using the formulae below:

1 $${\text{Absolute HRH gap }} = {\text{ current number }}{-}{\text{ required number}}{.}$$

This provided the actual number of understaffing or overstaffing of a particular cadre of staff in a health facility. A positive gap indicated overstaffing, whereas a negative gap indicated understaffing.

### Establishing relative HRH gaps (Staff Availability Ratio, SAR)

This measures the current staffing level (of a cadre of staff) as a ratio of the required number of staff as per the staffing norms.

2 $${\text{SAR}} = \frac{{\text{Current number of HRH}}}{{\text{Required number of HRH based on staffing norms}}}.$$

The SAR shows the amount of work pressure on the current staff, and its interpretation is similar to that of WISN ratio [31]. SAR of 1 indicates sufficient staff or an optimal staffing level. However, SAR less than 1 shows understaffing whilst SAR greater than 1 indicates overstaffing in the facility.

### Crude equity index

As a simplistic relative measure of equity in the national distribution of the health workforce, we computed a crude equity index which is a ratio of the Staff Availability Ratio in the best-staffed region to that of the worst-staffed region. This gives an indication of the number of folds the best-staffed region is better off than the worst-staffed region, and conversely the number of times (or folds) the worst-staffed region is worse off than the best-staffed region. However, it is limited in determining the degree to which each region has a fair share (or otherwise) of the national stock of the health workforce.

3 $${\text{Crude equity ratio}} = \frac{{{\text{Highest staff availablity ratio }}\left( {{\text{in the best - staffed region}}} \right)}}{{{\text{Lowest staff availablity ratio }}\left( {{\text{in the worst - staffed region}} } \right)}}.$$

### Costing of the staffing norms and gaps

A conservative approach was used in estimating HRH cost from the perspective of Central Government. The cost drivers included in the analysis were those budgeted for by the MoH/GHS and paid from the government’s consolidated fund. With this perspective, HRH cost incurred by local health facilities were not included. The main cost drivers considered for the analysis included:

- Gross annual salaries
- Gross market premium (this is an allowance paid to health workers supposedly in short supply)
- Other allowances (such as housing, on-call duty facilitation, fuel, utility).

There is salary differentiation between various grades within the staff categories. For this study, we used a weighted average method (WAM) to determine the weighted average salary of the staff category from the public sector salary scale of the Government of Ghana (GoG). The WAM was carefully selected having considered other alternative options such as the Simple Average of a category’s annual salary and starting salary of the first grade in each staff category. This method was most preferred as it is not affected by extreme salary values and staff numbers within a category. It sought to determine the relative staff grade numbers in each staff category vis-a-vis their respective corresponding annual salary per grade. The steps used were as follows:

The weight (*W*) in each category was determined by the number of staff on the grade (*N*) divided by the total number of staff (TN) in the category, mathematically expressed as

4 $$W \, = N/{\text{TN}}.$$

The weight (*W*) was then multiplied by the related staff cost (SC) resulting in the weighted staff cost (WSC) for the particular grade. The summation of the weighted staff cost for each grade determined the weighted average staff cost (WAC) for the category. These are expressed as follows:

5 $${\text{SC }} = {\text{ annual salary }} + {\text{ market premium}} + {\text{ other selected allowances drawn from the Gov't Consolidated Fund,}}$$

6 $${\text{WSC }} = \, W \, \times {\text{ SC}},$$

7 $${\text{WAC }} = \, \sum {\text{WSC}},$$

8 $${\text{Expected staff cost of a category }}\left( {{\text{EC}}} \right) \, = {\text{ TN }} \times {\text{ WA}},$$

9 $${\text{Current staff cost }}\left( {{\text{CSC}}} \right) \, = {\text{ payroll cost of a category,}}$$

10 $${\text{HR cost variance }} = {\text{ EC - CC}}.$$

A negative HR cost variance depicted the cost of inefficiently distributed staff whilst a positive cost variance represented the cost of HRH shortfall (i.e. the amount of money needed to meet the minimum staffing requirement). It must be noted that where the HR cost variance is zero, then the minimum staffing requirement was deemed to have been met.

### Data sources

A 3-year trend (2015–2018) of outpatient and inpatient data, used as proxies for workload or service utilisation, was extracted from the District Health Information Management System version 2 (DHIMS-2), the health data repository in Ghana. The health sector staffing norms classify health facilities according to their workload levels (using outpatients and inpatients as proxies) for HRH allocation [19, 32]. Based on the staffing norms, the workload data were used to identify the staffing requirements of each health facility in the GHS for 2018. The current staffing levels (number of the various cadres) as of May 2018 in each health facility were obtained from the individual health facility managers during a nationwide HRH gap analysis exercise.

## Results

### Aggregate human resources for health (HRH) gaps by region

Based on the minimum staffing requirement of the health sector staffing norms, the gap analysis revealed that, in aggregate, 105,440 health workers were needed to meet the minimum staffing needs of the GHS as at May 2018. However, a total of 61,756 were accounted for by the various districts and health facilities as their health workforce at post. This meant a staffing gap or vacancies of 47,758 were unfilled at GHS. Thus, as at the end of May 2018, GHS had only 59% of its aggregate staffing requirement leaving a vacancy rate of 41%. These aggregate figures, however, varied widely across various categories of staff and geographical locations. Table 3 provides details on the aggregate HRH requirements and gaps for all regions.

**Table 3:** Aggregate HRH requirements and gaps by region (all staff categories)

Region	Total staff required (a)	Total at post (b)	Absolute HR gaps (c = a−b)	Staff availability ratio (SAR = b/a) (%)
Ashanti	13,730	7854	6209	57
Brong Ahafo	10,510	5009	5777	48
Central	8283	5366	3245	65
Eastern	14,627	6390	8579	44
Greater Accra	9317	8497	1041	91
Northern	12,716	9335	4233	73
Upper East	6643	4011	3038	60
Upper West	7606	3169	4757	42
Volta	10,800	5738	5490	53
Western	11,208	6387	5389	57
National	105,440	61,756	47,758	59
*Crude equity index (highest/lowest)*				*2.17*

On average, Staff Availability Ratio for administrative and support staff was 54% as compared to 28% for allied health staff and 49% for clinical staff. See Additional file 2: Table S1 for details of the cadre-by-cadre requirements and gaps.

The results depicted in Table 3 show Greater Accra region as having the highest Staff Availability Ratio (SAR) of 91% followed by the Northern region with SAR of 73% and the Upper West Region with the lowest SAR of 42%. Of note, the crude equity index showed that in aggregate, the best-staffed region (Greater Accra) was 2.17 times (or 217%) better off than the worst-staffed region (Upper West).

### Descriptive analysis of staff availability across levels of health facilities

As shown in Table 4, the total number of staff required at the CHPS zones and compounds was about 14,670 (13.9% of the overall staffing requirements), while those currently at post were 10,082 representing SAR of 68.7%. Thus, about 7141 additional staff were required to fill the staffing gaps or vacancies at the CHPS level. About 827 out of 3,584 (23.1%) CHPS included in the analysis met the minimum staffing requirement of at least one (1) midwife at post while 3.2% (*n* = 113) of CHPS had the maximum staffing requirement of two (2) midwives as per the national policy (see Additional file 2). Approximately 74% of the CHPS Zones and Compounds failed to meet the minimum staffing requirement of at least one (1) midwife.

**Table 4:** HRH requirements and gaps for various levels of service delivery

Type of health facility	Total staff required (a)	Total at post (b)	Total HR gaps (c = a–b)	Staff availability ratio (SAR = b/a)
CHPS	14,670	10,082	7141	68.7%
Health Centre	29,521	15,357	14,419	52.0%
Polyclinic	4211	3333	879	79.1%
Primary Hospital	45,068	24,817	21,094	55.1%
Regional Hospital	050	5505	1679	78.1%
District Health Directorate^a^	3390	1456	2048	42.9%
Municipal Health Directorate^a^	1371	701	744	51.1%
Metropolitan Health Directorate^a^	162	70	93	43.2%
National	105,443	61,321	48,097	58.2%

^a^These are management and administrative structures overseeing the operations of health facilities and other public health interventions within their jurisdictions. Staff in these structures were included for the comprehensiveness of the analysis

Furthermore, against a staffing standard of a minimum of two (2) and a maximum of four (4), about 44% (1,572 out of 3,581) of CHPS had only one (1) Community Health Nurse (CHN) assigned to the zone while additional 46% of CHPS had at least two (2) CHNs. Overall, 3,350 representing 93.5% of the number of CHPS included in this analysis had at least one (1) CHN. Of the 11,627 CHNs accounted for in this analysis, 6,150 (53%) were deployed at CHPS while 3,763 (32%) were placed at Health Centres with responsibilities for outreach services.

Also, Health Centres required a total of 29,521 staff (28.0% of the overall staffing requirements) as compared to the current state where only 15,357 staff were available at post (52.9% SAR). Consequently, about 14 419 additional staff are required at Health Centres for optimal service delivery at this level. Particularly, only 47.1% (415 out of 882) of health centres had at least one (1) Physician Assistant, and even more critically, just 5.8% (*n* = 51) of health centres had up to two (2) Physician Assistants. However, 90.8% of all Health Centres had at least one (1) midwife at post, leaving 9.2% of health centres without midwives.

While about 4211 staff were required at Polyclinics, some 3333 staff were at post at the time of the gap analysis (representing SAR of 79.1%), leaving a staffing deficit of 879. Similarly, Primary Hospitals required about 45,068 staff (which representing 42.7% of the overall staffing requirements) as compared to 24,817 that were at post (SAR of 55.1%). Finally, Regional Hospitals needed a total of 7050 compared with 5,505 that were at post depicting SAR of 78.1%, and a staffing gap of 1679 across all the Regional Hospitals.

All primary (district) hospitals had at least one General Practitioner (Medical Officer). This means that the so-called ‘no man stations’ or ‘hospitals with no doctor’ had been eliminated by 2018. However, 16 hospitals (12.6%) were still *‘one-man stations”* and only 59.1% of the hospitals, mostly in urban areas, had 3 or more General Practitioners. Also, 87.5% of the primary hospitals had at least 10 midwives at post, whereas 92.2% of the hospitals recorded 15 or more General Nurses at post. However, just 61.7% of the hospitals had up to 35 or more General Nurses. Also, a paltry 36 out of 127 Primary Hospitals (28.3%) had Obstetrician & Gynaecologist at post.

Each Regional Hospital had at least two (2) Obstetricians & Gynaecologists at post although only 3 Regional Hospitals had 3 or more Obstetrics & Gynaecology specialists. Similarly, fewer than a quarter of the primary hospitals (24.4%, *n* = 31) had specialist surgeons while 18.1% of Primary hospitals and 90% of Regional Hospitals had Paediatricians at post. There was no primary or secondary hospital with a Dermatologist while only one (1) Regional Hospital had a Psychiatrist.

Regarding Critical Care Nurses (CCNs), less than a third of primary hospitals (27.3%, *n* = 35) were found to have at least one Critical Care Nurse at post. Thus, Critical Care Nurses were completely unavailable in over 70% of primary hospitals. Similarly, two (2) Regional Hospitals also lacked Critical Care Nurses. Also, 43.3% (*n* = 55) of primary hospitals had qualified Peri-operative Nurses. As low as 22% (*n* = 28) of primary hospitals and 60% (*n* = 6) of Regional Hospitals had trained Emergency Nurses. However, only 13 primary hospitals (10%) had up to two (2) trained Emergency Nurses.

### Aggregate HRH cost estimates: requirements, deficits and distributional inefficiencies

Using the Ministry of Health (MOH) and Ghana Health Service (GHS) perspective, the estimated cost (which included salaries, market premium and other allowances paid from the consolidated fund), of meeting the minimum staffing requirements was estimated to be about GH¢2,358,346,472 which is equivalent to US$521,758,069 (using December 2017 Interbank Exchange Rate of US$1: GH¢4.52) while the current cost of the staff at post was estimated at GH¢1,424,331,400 (US$315,117,566). The GHS, therefore, required an additional budget of GH¢1,335,069,404 (US$295,369,337) to meet the minimum requirement of staffing for the various levels of service delivery (see Table 5). This represented about 57% additional budgetary requirement to fill vacant posts to meet the minimum nationally agreed staffing norms.

**Table 5:** Cost of aggregate HRH requirements, gaps and inefficient distribution in Ghana by regions

Region	Total expected cost		Total current cost		Cost of inefficient staff distribution		Total cost of shortage		Proportion of inefficiency to current cost (%)
	(GH¢)	US$	(GH¢)	US$	(GH¢)	US$	(GH¢)	US$	
Ashanti	307,673,077	68,069,265	184,695,059	40,861,739	53,274,031	11,786,290	176,252,049	38,993,816	29
Brong Ahafo	232,105,773	51,350,835	114,796,453	25,397,445	23,396,508	5,176,219	140,705,828	31,129,608	20
Central	182,776,572	40,437,295	116,987,137	25,882,110	31,458,889	6,959,931	97,248,323	21,515,116	27
Eastern	322,420,490	71,331,967	146,622,828	32,438,679	23,017,244	5,092,311	198,814,906	43,985,599	16
Greater Accra	216,834,126	47,972,152	211,487,566	46,789,285	79,539,377	17,597,207	84,885,937	18,780,075	38
Northern	290,656,714	64,304,583	217,152,425	48,042,572	78,038,383	17,265,129	151,542,672	33,527,140	36
Upper East	148,811,404	32,922,877	92,074,647	20,370,497	24,167,133	5,346,711	80,903,890	17,899,091	26
Upper West	171,496,126	37,941,621	69,139,832	15,296,423	18,871,455	4,175,101	121,227,749	26,820,298	27
Volta	235,851,965	52,179,638	127,988,154	28,315,963	29,751,003	6,582,080	137,614,814	30,445,755	23
Western	249,720,225	55,247,837	143,387,300	31,722,854	39,540,309	8,747,856	145,873,235	32,272,840	28
National	2,358,346,472	521,758,069	1,424,331,400	315,117,566	401,054,332	88,728,835	1,335,069,404	295,369,337	28

However, in some health facilities, mostly in urban areas, it was observed that they had been staffed beyond the stipulated numbers in the staffing norms and could theoretically be deemed as inequitably distributed. The cost of this prevailing staff maldistribution across regions, districts and facilities (inefficient distribution of staff) was estimated at GH¢401,054,332 (US$88,728,835) annually. This represented 28.2% of the government’s expenditure on the wage bill. It was observed that the prevailing cost of inefficient staff distribution in the Greater Accra Region was about GH¢79,539,377 (US$17,597,207.30) which was the highest among all the regions. The region’s staffing cost based on numbers at post was estimated to be GH¢211,487,566 ($46,789,285) compared with the expected staffing cost (based on the staffing norms) of GH¢216,834,126 ($3,724,364), leaving inefficient staff distribution cost in the region of about 39% of its wage bill. Therefore, the potential efficiency savings from staff redistribution could offset the minimal staffing deficit if re-deployment of staff was pursued from areas of excess to areas of need in the region.

Also, the Northern and Ashanti regions were plagued with similar patterns of the high cost of inefficient staff distribution. For instance, while the Northern region was usually considered to be grossly understaffed, the analysis revealed that 36% of current staffing cost in the region was attributable to inefficient distribution which conservatively costs the government some GH¢78,038,383 ($17,265,129) annually. In the same vein, 29% of the prevailing staffing cost in the Ashanti region was attributable to inefficient distribution which amounted to potential efficiency savings of GH¢53,274,031 ($11,786,290.04) annually if redistribution was pursued in the region.

It is noteworthy, however, that the Eastern Region had the lowest cost of inefficient staff distribution of GH¢23,017,244 ($5,092,310.62) per annum which translated into 16% of the prevailing staffing cost. The region was, however, confronted with a significant shortage of health workforce which required a net investment of GH¢198,814,906 ($43,985,598.67) over 5 years after the re-deployment of excess staff to meet their minimum staff requirement. See Table 4 for details of the expected staffing cost compared with the current cost and the potential efficiency savings that could accrue from staff redistribution across all regions.

It is worth noting, however, that the aforesaid costs varied significantly across different categories of health workers as presented in a national summary in the Additional file 2: Table S1.

### Cost estimates of staffing requirements, gaps and inefficient distribution at various levels of service delivery

The analysis revealed significant health workforce expenditure gaps and inefficiencies for the various levels of healthcare delivery within the GHS (see Table 6). In particular, the expected annual staffing cost for CHPS was estimated at GH¢231,823,176 (US$51,288,313) as against prevailing expenditure of GH¢191,693,352 ($42,410,033)—21% less than optimal wage bill related expenditure at the community level. However, despite this apparent workforce expenditure deficit, the total cost of inefficient staff distribution within CHPS was estimated to be GH¢95,147,366 ($21,050,302.21) annually. Thus, almost 50% of prevailing staffing cost at CHPS could be optimised via possible staff redistribution especially for Community Health Nurses of which about 2992 could be redistributed to cover 77% of the existing gaps for Community Health Nurses.

**Table 6:** Cost estimates for staffing requirements, gaps and inefficient distribution by type of health facility

Type of facility	Total expected cost		Total current cost		Total cost of inefficient distribution		Total cost of shortage		% of inefficiency to current cost
	GHc	US$	GHc	US$	GHc	US$	GHc	US$	
CHPS	231,823,176	51,288,313	191,693,352	42,410,034	95,147,366	21,050,302	135,277,190	29,928,582	50%
Health Centres	570,040,243	126,115,098	320,121,782	70,823,403	100,744,215	22,288,543	350,662,676	77,580,238	31%
Polyclinics	98,443,018	21,779,429	80,600,579	17,831,987	30,218,681	6,685,549	48,061,119	10,632,991	37%
Primary Hospitals	1,163,063,130	257,314,852	634,088,004	140,284,957	120,239,721	26,601,708	649,214,847	143,631,603	19%
Regional Hospitals	197,989,928	43,803,081	149,837,220	33,149,827	40,162,967	8,885,612	88,315,674	19,538,866	27%
District Health Directorates	71,349,138	15,785,208	27,744,496	6,138,163	3,901,849	863,241	47,506,490	10,510,285	14%
Municipal Health Directorates	28,256,462	6,251,430	13,683,188	3,027,254	3,027,632	669,830	17,600,906	3,894,006	22%
Metropolitan Health Directorates	3,417,796	756,150	1,592,694	352,366	336,300	74,403	2,161,401	478,186	22%
National	2,364,382,891	523,093,560	1,419,361,317	314,017,990	393,778,730	87,119,188	1,338,800,304	296,194,758	28%

For Health Centres, the annual expected cost of staffing based on the staffing norms was estimated at GH¢570,040,243 (US$126,115,098) while the prevailing cost was estimated to be GH¢320,121,782 (US$70,823,403), but the prevailing cost also contained inefficient staff distribution which was roughly GH¢100,744,215 (US$22,288,543), representing 31% of the prevailing staffing expenditure. Therefore, the total cost of staff shortage could be ameliorated by about 29% if staff rationalisation is undertaken.

Furthermore, the annual expected cost of staffing Polyclinics was about GH¢98,443,018 (US$21,779,429) compared with a prevailing expenditure of GH¢80,600,579 ($17,831,987). However, maldistribution of staff amongst Polyclinics costs the taxpayer GH¢30,218,681($6,685,549) per annum, representing 37% of prevailing staffing cost. For Primary Hospitals, the total expected cost of staffing was estimated at GH¢1,163,063,130 (US$257,314,852) per annum, whereas the prevailing cost of staffing was about GH¢634,088,004 (US$140,284,956.64) of which GH¢120,239,721($26,601,708.19) or 19% was attributed to inefficient staff distribution.

Similarly, Regional Hospitals required an overall staffing cost of GH¢197,989,928 (US$43,803,081) compared to the prevailing expenditure of GH¢149,837,220 (US$33,149,827). About 27% (GH¢40,162,967 or $8,885,612) of the current cost was, however, attributable to inequitable distribution of staff which could offset nearly half of the additional staffing expenditure (GH¢88,315,674 or $19,538,866) needed across all the Regional Hospitals.

From the foregoing, it was apparent that while current staffing expenditure is generally below expected levels, an average of 22% (range: 14–50%) of existing staffing cost across the levels of primary and secondary healthcare could be better optimised using staff rationalisation.

## Discussion

Our analysis showed that as at the end of May 2018, GHS had only 59% of its aggregate staffing requirement leaving a vacancy rate of 41%. However, vacancy rates varied across levels of service delivery, cadres of staff and geographical locations. Results showed better staffing at the Polyclinics and Regional hospitals. Similar trends were also recorded when comparing administrative staff with clinical staff and urban areas to rural regions. Results point to both absolute and relative gaps in staffing in the Ghana Health Service. This picture of inequitable distribution of HRH within the Ghanaian context is in tandem with the long-standing picture in the majority of sub-Saharan Africa countries, where the preference for postings is skewed in favour of urban settings and higher level health facilities [3, 33].

There was, however, a gap in financing HRH as well as inefficiencies in utilisation of the wage bill as evidenced in the cost of the inequitable distribution. The wage bill cost of inequitable distribution was an area that had not been explored extensively as various measures to reduce the gap in the wage bill. In our analysis inefficiencies in spending ranged from 16 to 38% across the different geographical regions and from 14 to 50% across the different levels of health facilities. Although our analysis focussed solely on HRH, inefficiencies in resource use, in general, has been demonstrated in Ghana’s health sector where up to 65% of health facilities were found to be technically inefficient as they relatively used more resources than required for their level of output [20, 23, 24, 34]. Others have argued that health centres alone could save some US$7,062 annually [24], which could yield at least US$6 million efficiency savings per annum.

The crude equity index, which measured distributional disparity between the best-staffed region to the worst-staffed region was 2.17. This meant that the worst-staffed region (Upper West) was more than twice worst off than the best-staffed region (Greater Accra). This level of disparity in staffing situation between regions signalled high levels of inefficient staff distribution. It would, thus, be necessary to progressively monitor this indicator to track the impact of the implementation of the staffing norms and standards.

The analysis also showed the HRH gaps for the regions ranged from 1041 in the Greater Accra region to as high as 8579 in the Eastern region, giving a Staff Availability Ratio of 91% and 44%, respectively. The 91% Staff Availability Ratio of the Greater Accra region corresponded to international targets of at least 70% of nationally determined requirements [35]. The national picture of the HRH gaps stood at 47,708 with a Staff Availability Ratio of 59%. Thus, much needs to be done in terms of improving training outputs and recruitment to meet health workforce targets. Additionally, only polyclinics and regional hospitals were observed to have at least 70% of their staffing requirements with polyclinics having a Staff Availability Ratio of 79.1% and regional hospitals having 78.1%. Most polyclinics are located in urban areas so as the regional hospitals, which partly explains their relatively better staffing situation. On the other hand, the CHPS level which on average had 68.7% of their staffing requirement is one flagship area of the primary health care system in Ghana where substantial investment is being made [36]. Nonetheless, it suggests to some degree, the success of the CHPS policy in Ghana.

An amount of GH¢ 1,335,069,404 was required by the GHS to address the minimum staffing requirements for the various levels of service delivery, whilst the cost of current maldistribution across regions, districts and facilities stood at about GH¢ 401,054,332 representing 28.2% of current expenditure on staff at post. Regionally, the Greater Accra Region, which is found to have the highest cost associated with inequitable distribution, has also been cited in previous studies for a relatively very level of health workforce productivity [26]. In contrast, the Eastern Region was seen to be the region with the lowest cost of inequitable staff distribution needed a net investment of GH₵198,814,906 to address the HRH gaps in the region over the next 5 years.

Notwithstanding these findings, the study has a number of limitations. First, the analysis dealt with only one agency (the GHS) of the health sector in Ghana, which accounts for some 65% of the public sector health workforce [37]. Therefore, the analysis provides a tip of the iceberg rather than a comprehensive view of the health workforce inequity in Ghana. For instance, it is well documented that nearly half of doctors in the public sector were congregated at two of the biggest teaching hospitals which were not part of this analysis [38]. Owing to increasing demand for curative services, some CHPS facilities were found to be operating at the level of Health Centres whiles others were said to have been converted to health centres with Physician Assistants assigned to them—against the stated CHPS policy of the government [4]. Similarly, some urban polyclinics were also found to be operating at the level of primary hospitals with medical specialists, although they had not been officially designated to operate at those levels. These may have contributed to the need for higher levels of staffing in those facilities thereby increasing the magnitude of the inequitable distribution, but the lack of qualitative explanation to shed light on these should be considered a shortcoming of the analysis.

Another key limitation of this analysis is the quality and timeliness of the data used. The staffing data used in the analysis were manually obtained from stand-alone datasets of health facilities at a time there was no real-time integrated human resource information system which would have affected the data quality and comprehensiveness. For instance, there might have been staff movements (transfers between health facilities and regions) that took place soon after data collection and before the data analysis that were not accounted for. Also, the workload data which were taken from DHIMS-2 are known to have about 5% data incompleteness even though its timeliness is reportedly 100%. Finally, the crude equity index presented in this paper provides an aggregate level of geographical equity in the distribution of health workforce (between best and worst regions), but does not address the extent to which each region has a fair share or otherwise of the health workforce. Thus, the use and/or interpretation of the index should be done with caution.

## Conclusions, policy implications and impact

The evidence presented shows that despite the tremendous strides made to increase health workforce stock, the Ghana Health Service aggregately lacked at least 41% of its required staff in 2018. The challenges of health workforce shortages are often exacerbated by distributional disparities. However, the cost associated with both challenges and inequitable distribution has been seldomly estimated to inform policy. Consistent with previous works from an efficiency perspective, this analysis provides new insights that some 22% of the wage bill of Ghana Health Service is spent on health workers who are inequitably distributed if the health sector staffing norms of Ghana is used as the benchmark for distribution. Although the levels of health workforce budgetary deficits were as much as 57% on average, the cost of the inequitably distributed health workers could offset this budgetary deficit by almost 30%. Investing in the employment of trained, but unemployed health workers [39] and using evidence for health workforce planning and policies are prerequisites for addressing the aforesaid challenges. This analysis may only be a tip of the iceberg, hence a comprehensive health labour market analysis is imperative for holistic insights.

The analysis provided insights into several policies and implementation issues that could be addressed to improve the overall staffing situation and equity within the Ghana Health Service. It made a substantial contribution to ongoing health workforce planning transformation within the GHS. These included:

- *Improved budgetary allocation for recruitment of health workers *The overall shortfall in health worker availability was estimated at 61,900, but some 14,142 were also attributed to maldistribution, constituting 23% of the national shortfall. Thus, the net shortfall in staffing (if redistribution were to be made) was projected to be 47,758 across all categories of staff. This evidence was presented as part of the 2019 national budget planning process, which contributed to the allocation of additional recruitment for the health sector in 2019 culminating in the recruitment of 13,271 unemployed health workers [40]. However, this evidence-based planning needs to be further strengthened and sustained.
- *Improving health workforce information* To ensure sustainability in the monitoring and analysis of the health workforce distribution and equity, a Human Resource Information and Management System, HRIMS (https://www.ghsnewhrims.org/) was developed and deployed within the context of the National Health workforce Account (NHWA) [41] which at the time of writing this paper had a data completion rate of over 90%. The staffing norms and the gap analysis have been integrated into the HRIMS to sustainably repeat the analysis annually for decision-making on a real-time basis.
- *Redistribution strategy *Of the 14,142 staff that were deemed to be inequitably distributed, 11,600 (82%) constituted intra-regional distortions which required district- and regional-level redistribution. Only 2542 (18%) of the maldistribution was inter-regional in nature and required headquarters-led or national-level redistribution. Following extensive stakeholder deliberations, a draft redistribution concept was adopted, costed and the possible efficiency gains analysed over 5 years (this section is being reported in a separate paper).

For future recruitments and/or postings, priority should be given to Region, Districts and Facilities with the least staffing requirement as per the staffing norm to ensure that they meet their minimum staffing requirements. Consequently, a moratorium should be placed on postings to regions/facilities with optimal staffing requirement (except for replacement of disengaged staff) until there was relative equity in the HRH situation across regions/facilities. To facilitate this, recruitment processes were made online with each region given a quota based on the workforce gaps identified in the analysis. The Public Services Commission of Ghana also adopted the health sector staffing norms as the official establishment/staffing ceiling for health facilities. Although this is largely a cost-containment measure, it could contribute to addressing the inequitable distribution of health workers.

- *Health workforce production (training and education) *Although this analysis did not include supply-side analysis of the health workforce, the results, taken alongside previous works [12], paint a picture of education market failure where the current production of HRH seemed not to be matching health workforce need. For instance, there is the above-optimal availability of staff cadres such as auxiliary nurses or Enrolled Nurses (SAR:139%), Dental Prosthesis Technicians (SAR: 200%), IT Managers (SAR: 660%), Public Health Officers-Nutrition (SAR: 220%) and Opticians (SAR: 380%). It would thus, be imperative for a comprehensive health labour market analysis to engender evidence-based policy dialogue at the highest level to correct the current education market failure in favour of the production of staff cadres high in demand but currently short in supply. The current situation(s) of only 24% of hospitals having Specialist Surgeons, 82% of Primary Hospitals having no Paediatricians and all primary hospitals lacking appropriately qualified Emergency Medicine Physician and Psychiatrist could easily be corrected if the norms were to inform the production of the health workforce.
- *Leveraging task-sharing *Given that about 90% of the government’s subvention to the public health sector goes into the payment of employee compensation, the fiscal space for additional recruitment of HRH is increasingly constrained. As a result, GHS Leadership could consider deepening its already adopted task-sharing approach, which allows middle- and lower-level health professionals to assume duties and activities hitherto not part of their traditional roles. Allowing these middle- and lower-level health professionals to perform hitherto untraditional roles, of course with additional training, will bring about a rapid expansion of access to essential healthcare services, increase efficiency, and reduce health worker training and wage bill costs [42].

## Supplementary Information

**Additional file 1: Table S1.** Staffing Norms for CHPS. **Table S2.** Staffing Norms for Health Centres. **Table S3.** Staffing Norms for Polyclinics. **Table S4.** Categorisation of Primary Hospitals. **Table S5.** Staffing Norms for Primary Hospital. **Table S6.** Staffing Norms for Regional Hospitals. **Table S7.** Staffing Norms for Teaching Hospitals. **Table S8.** Staffing Norms for District/Municipal/Metropolitan Health Directorates. **Table S9.** Staffing Norms for Regional Health Directorates. **Table S10.** Staffing Norms for Health Training Institutions.

**Additional file 2: Table S1.** HRH Requirements, Gaps and Cost Estimates by Category of Staff, June 2018

## Data Availability

The datasets supporting our conclusions are publicly available and will be provided upon request.

## Abbreviations

CHPS: Community-based Health Planning and Services
CHN: Community Health Nurse
HRH: Human Resource for Health
GHS: Ghana Health Service
HWF: Health Workforce
MOH: Ministry of Health
SAR: Staff Availability Ratio
SDGs: Sustainable Development Goals
UHC: Universal Health Coverage
WHO: World Health Organization

## References

[1] Bossert TJ, World Health Organization, editors. Assessing financing, education, management, and policy context for strategic planning of human resources for health. Geneva, Switzerland: World Health Organization; 2007.

[2] Anand S, Bärnighausen T. Human resources and health outcomes: cross-country econometric study. The Lancet. 2004 (cited 2016 Oct 10);364:1603–1609. http://www.sciencedirect.com/science/article/pii/S0140673604173133 10.1016/S0140-6736(04)17313-3 15519630

[3] Munga MA, Mæstad O. Measuring inequalities in the distribution of health workers: the case of Tanzania. Hum Resour Health. 2009 (cited 2016 Oct 10);7. http://human-resources-health.biomedcentral.com/articles/10.1186/1478-4491-7-4 10.1186/1478-4491-7-4 PMC2655278 19159443

[4] MOH. National Community-Based Health Planning and Services (CHPS) Policy: Accelerating attainment of Universal Health Coverage and bridging the access inequity gap. Ministry of Health, Ghana; 2015.

[5] MOH. Health sector medium term development plan (HSMTDP), 2018–2021. Ministry of Health, Ghana; 2017.

[6] MOH. Health Sector Holistic Assessment of the Health Sector Performance-2015. Ministry of Health, Ghana; 2016.

[7] MOH. The Health Sector Medium-Term Development Plan, 2014 -2017. Ministry of Health, Ghana; 2014. www.moh.gov.gh.

[8] MOH. National Health Policy: Creating Wealth Through Health. Ministry of Health, Ghana; 2007 (cited 2016 Jun 4). http://www.moh-ghana.org/UploadFiles/Publications/NATIONAL%20HEALTH%20POLICY_22APR2012.pdf

[9] GHS. Human Resource Directorate Annual Report for 2017. Ghana Health Service, Human Resource Directorate; 2018.

[10] Campbell J, Buchan J, Cometto G, David B, Dussault G, Fogstad H, et al. Human resources for health and universal health coverage: fostering equity and effective coverage. Bull World Health Organ. 2013 (cited 2017 Jan 29);91:853–863. http://www.scielosp.org/scielo.php?pid=S0042-96862013001100853&script=sci_arttext 10.2471/BLT.13.118729 PMC3853950 24347710

[11] AHWO. Human Resources for Health Country Profile: Ghana, 2010. African Health Workforce Observatory; 2010.

[12] Asamani JA, Amertil NP, Ismaila H, Akugri FA, Nabyonga-Orem J. The imperative of evidence-based health workforce planning and implementation: lessons from nurses and midwives unemployment crisis in Ghana. Hum Resour Health. 2020 (cited 2020 Mar 6);18:16. https://human-resources-health.biomedcentral.com/articles/10.1186/s12960-020-0462-5 10.1186/s12960-020-0462-5 PMC7059310 32143724

[13] Asamani JA, Naab F, Ofei AMA. Leadership styles in nursing management: implications for staff outcomes. J Health Sci. 2016 (cited 2016 Apr 18);6:1–14. http://www.jhsci.ba/OJS/index.php/jhsci/article/view/266

[14] Scheffler RM, Mahoney CB, Fulton BD, Dal Poz MR, Preker AS. Estimates Of Health Care Professional Shortages In Sub-Saharan Africa By 2015. Health Aff (Millwood). 2009 (cited 2016 Jun 3);28:w849–62. http://content.healthaffairs.org/cgi/doi/10.1377/hlthaff.28.5.w849 10.1377/hlthaff.28.5.w849 19661111

[15] Asamani JA, Chebere MM, Barton PM, D’Almeida SA, Odame EA, Oppong R (2018). Forecast of healthcare facilities and health workforce requirements for the public sector in Ghana, 2016–2026. Int J Health Policy Manag.

[16] Dussault G, Franceschini MC. Not enough there, too many here: understanding geographical imbalances in the distribution of the health workforce. Hum Resour Health. 2006 (cited 2016 Aug 29);4:1. http://human-resources-health.biomedcentral.com/articles/10.1186/1478-4491-4-12 10.1186/1478-4491-4-12 PMC1481612 16729892

[17] Asamani JA. Equitable Access to a Functional Health Workforce in the Africa Region. 2016. http://www.afro.who.int/index.php?option=com_docman&task=doc_download&gid=10561&Itemid=2593

[18] MOH. Staffing Norms for the Health Sector. Ministry of Health, Ghana; 2018.

[19] WHO. Workload Indicators of Staffing Need (WISN): Selected Country Implementation Experiences. Hum Resour Health Obs Ser. 2016 (cited 2016 Jun 12);15. http://apps.who.int/iris/bitstream/10665/205943/1/9789241510059_eng.pdf

[20] Alhassan RK, Nketiah-Amponsah E, Akazili J, Spieker N, Arhinful DK, de Wit TFR (2015). Efficiency of private and public primary health facilities accredited by the National Health Insurance Authority in Ghana. Cost Eff Resour Alloc.

[21] Kirigia JM, Emrouznejad A, Cassoma B, Asbu EZ, Barry S. A Performance Assessment Method for Hospitals: The Case of. Effic Health Syst Units Afr Data Envel Anal. 2015. 10.1007/s10916-008-9157-5 19058655

[22] Novignon J, Nonvignon J (2017). Improving primary health care facility performance in Ghana: efficiency analysis and fiscal space implications. BMC Health Serv Res.

[23] Osei D, d’Almeida S, George MO, Kirigia JM, Mensah AO, Kainyu LH (2005). Technical efficiency of public district hospitals and health centres in Ghana: a pilot study. Cost Eff Resour Alloc.

[24] Dalaba MA, Welaga P, Matsubara C. Cost of delivering health care services at primary health facilities in Ghana. BMC Health Serv Res. 2017 (cited 2018 May 18);17. https://bmchealthservres.biomedcentral.com/articles/10.1186/s12913-017-2676-3 10.1186/s12913-017-2676-3 PMC5693519 29149853

[25] Appiah-Denkyira E, Herbst CH. Toward Evidence-Based Interventions for HRH. 2013 (cited 2017 Jan 29). http://elibrary.worldbank.org/doi/abs/10.1596/9780821396674_CH01

[26] Vujicic M, Addai E, Bosomprah S. Measuring Health Workforce Productivity: Application of a Simple Methodology in Ghana. 2009 (cited 2015 Dec 5). https://openknowledge.worldbank.com/handle/10986/13735

[27] MOH. Holistic Assessment of the Health Sector Programme of Work 2013. Accra: Ministry of Health, Ghana; 2014. http://www.moh.gov.gh/wp-content/uploads/2016/02/Holistic-Assessment-Report-June-2014140811072318.pdf

[28] Ghana Statistical Service. National population and housing census, year 2010 report. 2012;

[29] MOH. District Health Management Information System-Version 2 (DHIMS-2). Centre for Health Information Management (CHIM) -Ministry of Health; 2016. https://dhims.chimgh.org/dhims/dhis-web-commons/security/login.action;jsessionid=6F96FE74625334BE2AF90B42A05B840B

[30] MOH. Staffing Norms for the Health Sector of Ghana. Ministry of Health, Ghana; 2014. http://www.moh.gov.gh/wp-content/uploads/2016/02/2014-Summit-Staffing-Norm-Technical-Report-Revised.pdf.

[31] World Health Organization. Workload indicators of staffing need. 2010 (cited 2015 Oct 11); http://apps.who.int/iris/handle/10665/44414.

[32] MOH. Staffing Norms for the Health Sector of Ghana (Volume 1). Ministry of Health, Ghana; 2015.

[33] Ferrinho P, Siziya S, Goma F, Dussault G. The human resource for health situation in Zambia: deficit and maldistribution. Hum Resour Health. 2011 (cited 2015 Dec 4);9:30. http://www.biomedcentral.com/content/pdf/1478-4491-9-30.pdf 10.1186/1478-4491-9-30 PMC3283521 22182366

[34] Akazili J, Adjuik M, Jehu-Appiah C, Zere E. Using data envelopment analysis to measure the extent of technical efficiency of public health centres in Ghana. BMC Int Health Hum Rights. Springer; 2008;8:11. 10.1186/1472-698X-8-11 PMC2605432 19021906

[35] WHO. Global strategy on human resources for health: workforce 2030. World Health Organ. 2016. https://www.who.int/hrh/resources/globstrathrh-2030/en/.

[36] Nyonator FK, Awoonor-Williams JK, Phillips JF, Jones TC, Miller RA (2005). The Ghana community-based health planning and services initiative for scaling up service delivery innovation. Health Policy Plan Oxford University Press.

[37] GHS. Human Resource Annual Report—2018. Accra: Ghana Health Service; 2019.

[38] GHS. Human Resource Annual Report—2017. Accra: Ghana Health Service; 2017.

[39] Asamani JA, Amertil NP, Ismaila H, Akugri FA, Nabyonga-Orem J. The imperative of evidence-based health workforce planning and implementation: lessons from nurses and midwives unemployment crisis in Ghana. Hum Resour Health. 2020 (cited 2020 Mar 6);18:16. 10.1186/s12960-020-0462-5 10.1186/s12960-020-0462-5 PMC7059310 32143724

[40] GHS. Human Resource Annual Report—2019. Accra: Ghana Health Service; 2020.

[41] GHS. Human Resources Information and Management System (HRIMS). Ghana Health Service; 2020 (cited 2020 Apr 5). https://www.ghsnewhrims.org/Progress.php

[42] Padmanathan P, De Silva MJ (2013). The acceptability and feasibility of task-sharing for mental healthcare in low and middle income countries: a systematic review. Soc Sci Med Elsevier.

