# Supplementary material for: The cost of health workforce gaps and inequitable distribution in the Ghana Health Service: an analysis towards evidence-based health workforce planning and management
Source: Hum Resour Health. 2021 Mar 31;19:43. doi: 10.1186/s12960-021-00590-3 (PMC8010987; doi:10.1186/s12960-021-00590-3)
Supplement: Supplementary file 2 — Additional file 2: Table S1. HRH Requirements, Gaps and Cost Estimates by Category of Staff, June 2018 [file 12960_2021_590_MOESM2_ESM.doc]

**
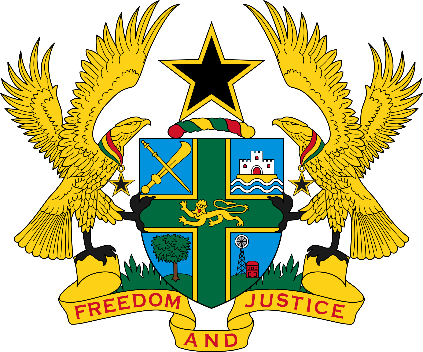
**

**MINISTRY OF HEALTH**

**STAFFING NORMS FOR THE HEALTH SECTOR IN GHANA**

***ABRIDGED VERSION***

**MARCH, 2018**

***SUPPORTED BY***


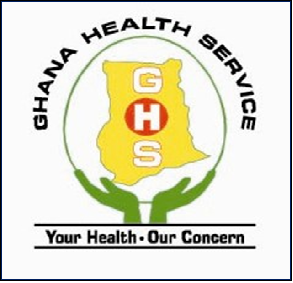

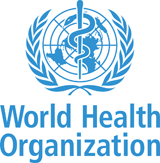

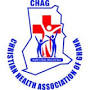

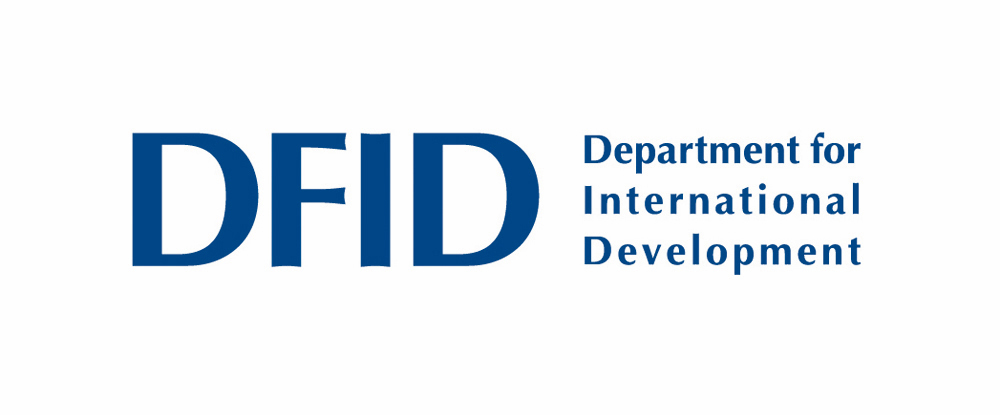


TABLE OF CONTENT

[**LIST OF TABLES** 1](#__RefHeading___Toc514314762)

[**ACKNOWLEDGEMENT** 2](#__RefHeading___Toc514314763)

[**MEMBERS OF THE STEERING COMMITTEE** 3](#__RefHeading___Toc514314764)

[**WISN STUDY AND DATA COLLECTION TEAM** 4](#__RefHeading___Toc514314765)

[**TECHNICAL WORKING GROUP** 5](#__RefHeading___Toc514314766)

[**3.0 THE STAFFING NORMS** 6](#__RefHeading___Toc514314767)

[**3.1 INTRODUCTION** 6](#__RefHeading___Toc514314768)

[**3.2 STAFFING NORMS FOR CHPS** 7](#__RefHeading___Toc514314769)

[**3.3 STAFFING NORMS FOR HEALTH CENTRES** 8](#__RefHeading___Toc514314770)

[**3.4 STAFFING NORMS FOR POLYCLINICS** 9](#__RefHeading___Toc514314771)

[**3.5 STAFFING NORMS FOR PRIMARY HOSPITALS** 11](#__RefHeading___Toc514314772)

[**3.6 STAFFING NORMS FOR REGIONAL HOSPITALS** 15](#__RefHeading___Toc514314773)

[**3.7 STAFFING NORMS FOR TEACHING HOSPITALS** 19](#__RefHeading___Toc514314774)

[3.8 STAFFING NORMS FOR DISTRICT/MUNICIPAL/METROPOLITAN HEALTH DIRECTORATES 23](#__RefHeading___Toc514314775)

# LIST OF TABLES

Table 1: Staffing Norms for CHPS [7](#__RefHeading___Toc514314776)

Table 2: Staffing Norms for Health Centres [8](#__RefHeading___Toc514314777)

Table 3: Staffing Norms for Polyclinics [10](#__RefHeading___Toc514314778)

Table 4: *Categorisation of Primary Hospitals* [12](#__RefHeading___Toc514314779)

Table 4: Staffing Norms for Primary Hospital [13](#__RefHeading___Toc514314780)

Table 5: Staffing Norms for Regional Hospitals [16](#__RefHeading___Toc514314781)

Table 6: Staffing Norms for Teaching Hospitals [20](#__RefHeading___Toc514314782)

Table 7: Staffing Norms for District/Municipal/Metropolitan Health Directorates [24](#__RefHeading___Toc514314783)

Table 8: Staffing Norms for Regional Health Directorates [25](#__RefHeading___Toc514314784)

Table 9: Staffing Norms for Health Training Institutions [27](#__RefHeading___Toc514314785)

# ACKNOWLEDGEMENT

The Steering Committee and Technical Working Group wish to acknowledge with gratitude of members of the Health Sector Working Group (HSWG) and all the Senior Health Managers who conceived and greatly supported the development of staffing norms. For the completion of the 30% that were left in the volume 1, the TWG is particularly grateful for the efforts of the following persons.

Hon. Kweku Agyemang-Manu - Minster of Health

Dr Anthony Nsiah-Asare - Director-General, GHS

Dr John Koku Awoonor-Williams - Director, PPMED, GHS

Dr Margaret Chebere - Director, HRD, GHS

Dr Kwesi Asabir - Head, HTIs

We are particularly grateful to DFID and WHO for the enormous technical and financial support towards the completion of this document.

Finally, we wish to thank all staff who participated in the process especially at the health facility level.

# MEMBERS OF THE STEERING COMMITTEE

The Steering Committee provided policy direction and leadership for the implementation of the WISN process and the subsequent development of Staffing Norms from the WISN results. Members of the Steering Committee are:

1. Hon. Tina Mensah - Hon. Deputy Minister of Health
2. Dr Anthony Nsiah-Asare - Director-General, Ghana Health Service
3. Dr John Koku Awoonor-Williams - Director, PPMED, GHS
4. Mr Peter Yeboah - Executive Director, CHAG
5. Dr Alfred Sugri Tia - Former Hon. Deputy Minister of Health
6. Dr Afisah Zakariah - Chief Director, MOH
7. Madam Salamatu Abdul Salam - Former Chief Director, MOH
8. Dr Sylvester Anemana - Former Chief Director, MOH
9. Dr Ebenezer Appiah Denkyira - Former Director-General, GHS
10. Mr Alexander Arphul - Former Director, HRHD, MOH
11. Dr Erasmus Agongo - Former Director, PPME, GHS
12. Mr Herman Dusu - Former Financial Controller, MOH
13. Dr Margaret Chebere - Director, HRD, GHS
14. Dr Gilbert Buckle - Former Chief Executive Officer, KBTH
15. Mrs Ramatu Ude Umanta - Director, Finance, GHS
16. Mr Selassi Amah d’Almeida - Health Economics Advisor, WHO, Ghana
17. Mr Emmanuel Owusu Ansah - Former Acting Director, PPME, MOH

# WISN STUDY AND DATA COLLECTION TEAM

The team collected data from the selected health facilities and performed the facility-based WISN analysis. The team included:

1. Dr Margaret Chebere
2. Mr Victor Francis Ekey
3. Mr Selassi Amah d'Almeida
4. Mrs Georgina Yeboah
5. Mr James Avoka Asamani
6. Ms Anna Plange
7. Dr Kwasi Asabir
8. Mr Gabby Alex Hottordze
9. Mr Emmanuel Owusu Ansah
10. Mr Stephen Darko
11. Mr Francis Kyereboah
12. Mrs Gladys Amankwah
13. Mr Samuel Nugblega
14. Ms Ekui Dovlo
15. Dr Kwasi Yeboah-Awudzi
16. Mr Kojo Baisie
17. Mr Peter Obiri-Yeboah
18. Mr T. A. Mahmoud
19. Mr Molayo Decker
20. Mr Hamza Ismaila
21. Mr Karikari Marfo
22. Dr Kofi Gafatsi Normanyo
23. Dr Horlali Yao Gudjinu
24. Mr Zanu Dassah
25. Hajia Kubura Sulemana
26. Ms Elsie Okoh
27. Mr Jerry Kwame Asamoah
28. Ms Modesta Abbem
29. Mr Frank Delasi Amenorpe
30. Mr Ahmed Abdul-Majeed
31. All Regional HR Managers, Ghana Health Service

# TECHNICAL WORKING GROUP

The Technical Working Group (TWG) undertook further analysis of the facility-based WISN results to develop the Staffing Norms.

1. Dr John Koku Awoonor-Williams - Director, PPMED, GHS
2. Dr Margaret Chebere - Director, HRD, GHS
3. Dr Kwasi Asabir - Deputy Director & Head, HTI, MOH
4. Mr Selassi Amah d'Almeida - Health Economics Advisor, WHO
5. Mr Francis Victor Ekey - Deputy Director, HR Planning, GHS
6. Mrs Georgina Yeboah - Deputy Director, HRM, KATH
7. Mr James Avoka Asamani - Health Economist/Health Policy Specialist
8. Mr Peter Obiri-Yeboah - Deputy Director, HRM, GHS
9. Mr Francis Kyereboah - HR Planning Specialist
10. Mrs Gladys Amankwah - HR Administrator, CHAG
11. Ms Anna Plange - Head of Recruitment, GHS
12. Mr Stephen Darko - Head, HR Information, GHS
13. Mr Jerry Kwame Asamoah - HRD, GHS
14. Mr Frank Delasi Amenorpe - Snr. Nursing Officer, Atibie Gov’t Hospital
15. Mr Hamza Ismaila - Administrator, HRD, GHS
16. Ms Ekui Dovlo - Chief Statistician, HRD, GHS
17. Ms Modesta Abbem - Regional HR Manager, Ashanti
18. Mr Abdul-Majeed Ahmed - Health Economist/Health Policy Specialist
19. Mr Zanu Dassah - Deputy Director, Training & Dev’t, GHS
20. Ms Edith Mansah - Health Planner, PPMED, GHS

# 3.0 THE STAFFING NORMS

## 3.1 INTRODUCTION

Staffing Norm is a human resource planning and management tool that gives an indication of the number and calibre of staff required in a given health facility/institution. It is intended to facilitate efficient health workforce utilisation, a more equitable distribution and accountability.

This staffing norm is a workload-related evidence-informed one that is quite simple to use. It is designed to accommodate changes in workload levels from time to time. To use this staffing norm appropriately, one needs ***workload of health facilities taken from only reported statistics*** in reliable sources and databases such as DHIMS and annual reports. As much as possible, the use of unofficial data or anecdotal ‘evidence’ should be avoided. ***It is also better to use the average of a facility’s annual data for two or three consecutive years.***

Each health facility type is assigned a range of staffing requirements for each staff category. Health facilities should use the minimum staffing requirement as the basis for gap analysis and initial HR planning which should then be adjusted on annual basis using the adjustment/projection guide established in volume 1(see Appendix 1). Where the workload change far exceeds 15%, it is advisable to check if the workload category has changed or not.

**NOTE:** The staffing requirements given in this norm are in aggregates for staff categories and thus, Managers need to ensure an appropriate skill mix within the numbers based on staff availability and context (see Appendix 12 for guidelines on intra-category skill mix). It should be understood that the norm is a guide and does not replace managerial responsibility of critical analysis of the context. However, reasons to exceed the norms should always be based on ‘compelling evidence’.

## 3.2 STAFFING NORMS FOR COMMUNITY-BASED HEALTH PLANNING AND SERVICES (CHPS)

CHPS are categorized into A, B and C based on workload. Category A has an annual workload of 4,000 or less while category B has annual workload between 4,000 and 8,000. Category C, on the other hand, have annual workload exceeding 8,000. The staffing norm of each category is provided in Table S1 below.

**Table S1**: Staffing Norms for CHPS

| **CATEGORY** | **STAFFING REQUIREMENTS** | | | | | | **REMARKS** |
| --- | --- | --- | --- | --- | --- | --- | --- |
|  | **CATEGORY A**  **(also includes all CHPS with No Compounds)** | | **CATEGORY B** | | **CATEGORY C**  **(Facilities functioning at the level of Health Centre but designated as CHPS)** | |  |
|  | **MIN** | **MAX** | **MIN** | **MAX** | **MIN** | **MAX** |  |
| Community Health Officer/Nurse | 2 | 3 | 2 | 4 | 3 | 4 |  |
| Midwife | 0 | 0 | 1 | 2 | 2 | 2 | ***Only at facilities where full midwifery services are to be provided*** |
| Field Technician | 0 | 0 | 0 | 0 | 1 | 1 |  |
| Enrolled Nurse | 0 | 0 | 0 | 0 | 1 | 2 | ***Only in the facilities that are functioning at the level of Health Centres but designated as CHPS and the annual workload is more than 8,000.*** |
| Registered General Nurse | 0 | 0 | 0 | 0 | 1 | 1 |  |
| Security Guard | 1 | 2 | 1 | 2 | 1 | 2 | ***Only where there is a Compound*** |

## 3.3 STAFFING NORMS FOR HEALTH CENTRES

Health centres are categorized into two broad groups based on their annual outpatient coverage. The health centre with the least staff requirements classified as “A” needs to cater for at least 12,655 outpatients per year as against category “B” which attend to between 13,678 and 28,119 outpatients per year. Any health centre that attends to a minimum of 29,525 outpatients per annum qualifies for staffing requirements similar to that of polyclinic. For health centre classified in category “A” to move to a higher category of “B”, it needs to increase its outpatient coverage per annum by at least 5 percent as shown in Table 6 below.

**Table S2: Staffing Norms for Health Centres**

| **STAFF CATEGORY** | **WORKLOAD CATEGORY A** | | **WORKLOAD CATEGORY B** | | **REMARKS** |
| --- | --- | --- | --- | --- | --- |
|  | **Minimum** | **Maximum** | **Minimum** | **Maximum** |  |
| Finance Officer | 0 | 0 | 1 | 2 |  |
| Accounts Officer (cash & NHIS) | 1 | 1 | 1 | 2 |  |
| Biostatistics Assistant | 1 | 2 | 2 | 3 |  |
| Physician Assistant (Medical) | 1 | 2 | 3 | 4 |  |
| General Nurses | 3 | 6 | 7 | 13 |  |
| Enrolled Nurses | 3 | 5 | 5 | 8 |  |
| Midwives | 2 | 4 | 4 | 6 |  |
| Community Health Nurse | 4 | 8 | 8 | 12 |  |
| Mental Health Nurse | 1 | 2 | 2 | 3 |  |
| Field Technician | 1 | 1 | 1 | 2 |  |
| Laboratory Technician | 0 | 0 | 1 | 2 |  |
| Laboratory Assistant | 1 | 2 | 2 | 3 |  |
| Dispensing Technicians and/or Dispensing Assistant | 1 | 3 | 3 | 4 |  |
| Storekeeper | 1 | 1 | 1 | 2 |  |
| Community Mental Health Officer | 1 | 1 | 1 | 1 | Sub-district |
| Driver | 1 | 2 | 1 | 2 | *In accordance with the transport policy one driver per functional car* |
| Technical Officer (Health Information) | 1 | 1 | 1 | 1 | Sub-district |
| Technical Officer (Disease Control) | 1 | 1 | 1 | 1 | Sub-district |
| Technical Officer (Laboratory) | 1 | 1 | 1 | 1 |  |
| Technical Officer (Health Promotion) | 1 | 1 | 1 | 1 | Sub-district |
| Technical Officer (Nutrition) | 1 | 1 | 1 | 1 | Sub-district |
| Hospital Orderly | 1 | 2 | 2 | 4 |  |
| Labourer | 1 | 2 | 1 | 2 |  |
| Watchman | 1 | 2 | 1 | 2 |  |

*Note: Staff categories with ‘Sub-district’ remarks are to be placed at health centre designated as the Sub-district headquarters.*

## 3.4 STAFFING NORMS FOR POLYCLINICS

Using OPD attendance, Polyclinics are categorized as A or B. Category B polyclinics records at least 31,500 OPD attendance per annum whilst those in category A records 30,000 or less per annum. It is observed that Polyclinics with the workload in category B tends to be functioning at the level of Primary Hospitals and thus requires similar levels of staffing. However, they are NOT by virtue of this workload level automatically designated as Primary Hospitals. Such designation could require other considerations beyond workload and staffing needs. The staffing norms for policlinics is detailed in Table S3.

**Table S3**: Staffing Norms for Polyclinics

| **CATEGORY** | **CATEGORY A** | | **CATEGORY B** | |
| --- | --- | --- | --- | --- |
|  | **Annual OPD <30,000** | | **Annual OPD >31,500** | |
|  | **MIN** | **MAX** | **MIN** | **MAX** |
| Accountant | 1 | 1 | 1 | 2 |
| Accounts Officer | 3 | 4 | 5 | 6 |
| Finance Officer | 1 | 1 | 1 | 2 |
| Internal Auditor | 1 | 1 | 1 | 2 |
| Biomedical Scientist | 2 | 3 | 3 | 4 |
| Laboratory Assistant | 2 | 3 | 3 | 4 |
| Technical Officer (Laboratory) | 6 | 9 | 9 | 13 |
| Technical Assistant (Biostatistics) | 5 | 7 | 7 | 11 |
| Technical Officer (Biost/HI) | 2 | 3 | 3 | 4 |
| Medical Officer | 1 | 2 | 4 | 7 |
| Field Technician | 1 | 1 | 1 | 2 |
| Midwife | 5 | 7 | 6 | 11 |
| Community Health Nurse | 8 | 12 | 12 | 18 |
| Enrolled Nurse | 10 | 15 | 20 | 30 |
| General Nurse | 10 | 15 | 15 | 26 |
| ENT Nurse | 1 | 1 | 1 | 2 |
| Ophthalmic Nurse | 1 | 1 | 1 | 2 |
| Public Health Nurse | 1 | 2 | 2 | 3 |
| Nutrition Officer | 1 | 1 | 1 | 2 |
| Dispensing Assistant | 2 | 3 | 3 | 4 |
| Pharmacist | 1 | 2 | 2 | 3 |
| Pharmacy Technician | 3 | 4 | 5 | 6 |
| Physician Assistant (COHO) | 1 | 1 | 1 | 2 |
| Physician Assistant (Medical) | 3 | 4 | 4 | 8 |
| Mental Health Nurse | 1 | 1 | 1 | 2 |
| Radiographer | 1 | 1 | 1 | 2 |
| Health Service Administrator | 1 | 1 | 1 | 2 |
| Procurement Officer | 1 | 1 | 1 | 2 |
| Storekeeper | 1 | 1 | 1 | 2 |
| Supply Officer | 1 | 1 | 1 | 2 |
| Nutrition Technical Officer | 1 | 1 | 1 | 2 |
| Technical Officer (Disease Control) | 1 | 2 | 2 | 3 |
| Driver[[1]](#footnote-2) | 1 | 2 | 1 | 2 |
| Hospital Orderly | 3 | 4 | 6 | 9 |
| Labourer | 1 | 1 | 1 | 1 |
| Watchman/Security Guard | 2 | 3 | 4 | 6 |

## 3.5 STAFFING NORMS FOR PRIMARY HOSPITALS

District (primary) hospitals vary in terms of their workload levels. Using outpatients[[2]](#footnote-3) and admissions[[3]](#footnote-4) per annum as proxies, the district hospitals are categorized into four broad bands to facilitate determining their staffing requirements. District hospitals with the least staffing requirements classified as A are those that attend to a maximum of 46,574 outpatients and 4,157 admissions per annum. Category B are those that attend to between 48,903 – 76,308 outpatient cases and between 4,685 – 6,563 inpatient clients per annum whilst Category C outpatient cases range between 80,123 – 100,000 and admissions of 6,834 – 9,000 per annum. The category D which has the highest staff requirements should attend to at least 105,000 outpatients and 9,450 admissions per year. For a facility to move from one category to a higher one there is the need for it to increase its outpatient and admission outputs by at least 5 percent beyond its upper limit. For facility in category A to be reclassified as B, there it must increase its maximum outpatient output from 44,574 to 48,903 and its admissions from 4,157 to 4,685.

***Table S4:*** Categorisation of Primary Hospitals

| Total Annual OPD | Total Annual Admissions | | | |
| --- | --- | --- | --- | --- |
|  | ≤4157 | 4685 - 6563 | 6834 – 9000 | 9450 + |
| ≤46574 | A |  |  |  |
| 48903 – 76308 |  | B |  |  |
| 80123 – 100000 |  |  | C |  |
| 105000 + |  |  |  | D |

The staffing norms for the categories of health workforce that were not covered in volume is detailed in Table S5 below.

**Table S5: Staffing Norms for Primary Hospital**

| **STAFF CATEGORY** | **WORKLOAD CATEGORY A** | | **WORKLOAD CATEGORY B** | | **WORKLOAD CATEGORY C** | | **WORKLOAD CATEGORY D** | |
| --- | --- | --- | --- | --- | --- | --- | --- | --- |
|  | **Min.** | **Max.** | **Min.** | **Max.** | **Min.** | **Max.** | **Min.** | **Max.** |
| Accountant | 1 | 2 | 2 | 3 | 3 | 4 | 4 | 6 |
| Finance Officer | 2 | 3 | 2 | 4 | 5 | 8 | 7 | 12 |
| Accounts Officer | 5 | 7 | 9 | 11 | 13 | 18 | 16 | 22 |
| Internal Auditor | 1 | 1 | 1 | 1 | 1 | 2 | 1 | 2 |
| Finance Officer (Audit) | 1 | 1 | 1 | 1 | 1 | 2 | 2 | 3 |
| Biomedical Scientist | 3 | 4 | 4 | 6 | 6 | 7 | 8 | 12 |
| Technical Officer (Laboratory) | 7 | 10 | 9 | 15 | 12 | 18 | 15 | 22 |
| Laboratory Assistant | 5 | 8 | 7 | 10 | 10 | 14 | 12 | 20 |
| Biostatistics/Medical Records Assistant | 8 | 11 | 11 | 15 | 16 | 19 | 20 | 25 |
| Biostatistics Officer | 1 | 2 | 1 | 2 | 1 | 2 | 1 | 3 |
| Clinical Engineering Technologist | 1 | 2 | 2 | 3 | 2 | 3 | 2 | 3 |
| Clinical Engineering Manager | 0 | 1 | 0 | 1 | 1 | 1 | 1 | 2 |
| Dental Surgeon | 1 | 2 | 1 | 2 | 1 | 2 | 2 | 3 |
| Dental Surgery Assistant | 1 | 2 | 1 | 2 | 1 | 2 | 1 | 2 |
| Dental Technician | 1 | 2 | 1 | 2 | 1 | 2 | 1 | 2 |
| Medical Officers | 3 | 7 | 7 | 10 | 13 | 17 | 23 | 31 |
| Obstetrician & Gynaecologist | 1 | 2 | 2 | 3 | 3 | 5 | 5 | 8 |
| Ophthalmologist | 1 | 1 | 1 | 1 | 1 | 2 | 1 | 2 |
| Paediatrician | 1 | 2 | 2 | 3 | 3 | 4 | 4 | 6 |
| General Surgeon | 1 | 1 | 1 | 2 | 1 | 2 | 2 | 3 |
| Family Medicine Physician | 1 | 1 | 1 | 2 | 1 | 2 | 1 | 3 |
| Midwife | 10 | 29 | 30 | 41 | 43 | 52 | 55 | 84 |
| Community Health Nurse | 3 | 7 | 7 | 10 | 10 | 13 | 13 | 20 |
| Enrolled Nurses | 19 | 42 | 44 | 60 | 63 | 76 | 80 | 123 |
| Registered Nurse | 35 | 64 | 67 | 91 | 95 | 115 | 121 | 186 |
| Ophthalmic Nurse | 1 | 2 | 2 | 3 | 2 | 3 | 3 | 5 |
| Public Health Nurse | 2 | 4 | 4 | 6 | 6 | 7 | 8 | 12 |
| Pharmacist | 2 | 3 | 3 | 5 | 5 | 7 | 6 | 12 |
| Pharmacy Technician | 6 | 9 | 8 | 13 | 8 | 16 | 12 | 20 |
| Pharmacy Assistant | 3 | 5 | 4 | 6 | 5 | 8 | 5 | 12 |
| Certified Registered Anaesthetist | 2 | 3 | 3 | 5 | 5 | 8 | 8 | 13 |
| Physician Assistant (COHO) | 1 | 2 | 2 | 3 | 3 | 4 | 3 | 4 |
| Physician Assistant (Medical) | 2 | 3 | 3 | 4 | 4 | 5 | 6 | 9 |
| Physician Assistant (Herbal) | 1 | 1 | 1 | 1 | 1 | 2 | 1 | 2 |
| Mental Health Nurse | 2 | 3 | 3 | 4 | 4 | 5 | 4 | 6 |
| X-ray Technician | 2 | 3 | 3 | 4 | 4 | 5 | 6 | 9 |
| Radiographers | 2 | 3 | 2 | 4 | 4 | 5 | 5 | 9 |
| Technical Assistant (X-Ray) | 1 | 1 | 1 | 2 | 2 | 3 | 3 | 4 |
| Artisans (Mechanic, Electricals, Plumbing) | 6 | 10 | 11 | 14 | 15 | 18 | 19 | 29 |
| Health Service Administrator | 1 | 1 | 1 | 1 | 1 | 2 | 1 | 2 |
| Human Resource Manager | 1 | 1 | 1 | 2 | 1 | 2 | 1 | 2 |
| IT Manager | 1 | 2 | 1 | 2 | 2 | 4 | 4 | 6 |
| IT Officer/Technician | 2 | 3 | 2 | 3 | 3 | 4 | 5 | 7 |
| Procurement Officers | 1 | 1 | 1 | 2 | 2 | 3 | 2 | 3 |
| Procurement Manager | 1 | 1 | 1 | 1 | 1 | 2 | 2 | 2 |
| Supply Manager | 1 | 1 | 1 | 1 | 1 | 2 | 1 | 2 |
| Supply Officer | 1 | 2 | 1 | 2 | 2 | 3 | 2 | 4 |
| Blood Bleeder/Phlebotomist | 1 | 2 | 2 | 3 | 3 | 4 | 4 | 6 |
| Clinical Pharmacist | 1 | 1 | 1 | 2 | 2 | 4 | 3 | 7 |
| Blood Donor Organizer | 1 | 2 | 2 | 3 | 2 | 4 | 3 | 5 |
| Critical Care Nurse | 3 | 5 | 5 | 7 | 7 | 11 | 12 | 17 |
| Emergency Nurse | 8 | 12 | 13 | 16 | 17 | 20 | 21 | 30 |
| ENT Nurse | 1 | 2 | 1 | 3 | 2 | 3 | 3 | 5 |
| Peri-Operative Nurse | 5 | 8 | 8 | 11 | 11 | 13 | 14 | 20 |
| Public Health Nurse | 2 | 3 | 3 | 4 | 4 | 5 | 5 | 6 |
| Clinical Psychologist | 1 | 2 | 1 | 2 | 1 | 2 | 2 | 3 |
| Executive Officer | 2 | 2 | 2 | 3 | 2 | 3 | 2 | 4 |
| Dietician | 1 | 2 | 2 | 3 | 2 | 3 | 3 | 5 |
| Optician | 1 | 2 | 1 | 2 | 2 | 3 | 3 | 5 |
| Optometrist | 1 | 2 | 1 | 2 | 2 | 3 | 3 | 5 |
| Physiotherapist | 1 | 1 | 1 | 1 | 1 | 2 | 2 | 3 |
| Physiotherapy Assistant | 1 | 2 | 1 | 2 | 1 | 2 | 2 | 3 |
| Prosector | 1 | 1 | 1 | 1 | 1 | 1 | 1 | 1 |
| Technical Officer (Disease Control) | 1 | 2 | 2 | 3 | 2 | 3 | 3 | 5 |
| Catering Officer | 1 | 2 | 1 | 2 | 2 | 3 | 3 | 5 |
| Driver | 2 | 2 | 2 | 3 | 3 | 4 | 4 | 6 |
| Hospital Orderly | 12 | 13 | 13 | 17 | 18 | 21 | 22 | 32 |
| Labourer | 3 | 5 | 5 | 7 | 7 | 8 | 9 | 13 |
| Launderer | 3 | 4 | 4 | 5 | 6 | 7 | 7 | 10 |
| Mortuary Attendant | 1 | 2 | 1 | 2 | 2 | 3 | 3 | 5 |
| Nutrition Officer | 1 | 1 | 1 | 1 | 1 | 2 | 1 | 2 |
| Technical Officer (Nutrition) | 1 | 2 | 1 | 2 | 1 | 2 | 2 | 3 |
| Private Secretary | 1 | 2 | 1 | 2 | 2 | 2 | 2 | 3 |
| Security Guard | 5 | 8 | 8 | 11 | 11 | 13 | 14 | 20 |
| Staff Cook | 2 | 3 | 3 | 4 | 4 | 5 | 5 | 8 |
| Telephonist | 1 | 2 | 1 | 2 | 2 | 3 | 3 | 5 |
| Transport Officer | 1 | 2 | 1 | 2 | 1 | 2 | 3 | 4 |
| Field Technician | 1 | 2 | 1 | 2 | 1 | 2 | 2 | 3 |
| Administrative Manager | 1 | 2 | 1 | 2 | 1 | 2 | 2 | 3 |
| Technical Officer (Health Promotion) | 1 | 1 | 1 | 1 | 1 | 2 | 1 | 2 |
| Technical Officer (Health Information) | 1 | 2 | 2 | 3 | 3 | 3 | 3 | 5 |

## 3.6 STAFFING NORMS FOR REGIONAL HOSPITALS

Regional Hospitals were previously not categorised in first phase but have now been categorised into two, A or B based on workload. Annual OPD attendance and inpatients data from DHIMS-2 or other reliably reported sources (s) such as annual reports are used to determine the workload category. No difference in scope or range of services is implied or inferred in the categorization but just the workload volumes. The staffing norms for Regional Hospitals are shown in Table S6.

**Table S6**: Staffing Norms for Regional Hospitals

| **Category** | Category A | | Category B | |
| --- | --- | --- | --- | --- |
|  |  |  |  |  |
|  | OPD/Year below 100,000 and inpatient below 10,000 | | At least 110,000 OPD/Year and Inpatient of 15,000 | |
|  |  |  |  |  |
|  | Min | Max | Min | Max |
| Accountant | 2 | 3 | 4 | 6 |
| Accounts Officer | 5 | 10 | 11 | 21 |
| Finance Officer | 3 | 5 | 5 | 9 |
| Internal Auditor | 1 | 2 | 2 | 3 |
| Biomedical Scientist | 4 | 8 | 12 | 23 |
| Technical Officer (Laboratory) | 3 | 6 | 8 | 15 |
| Biostatistics Assistant | 4 | 8 | 8 | 16 |
| Biostatistics Officer | 1 | 1 | 2 | 3 |
| Technical Officer (Health Information) | 2 | 3 | 4 | 8 |
| Biomedical Engineer/Technologist | 1 | 2 | 2 | 3 |
| Dental Surgeon | 1 | 2 | 2 | 3 |
| Dental Surgery Assistant | 1 | 2 | 3 | 5 |
| Dental Technician (Prosthesis) | 1 | 1 | 1 | 2 |
| Maxillofacial Surgeon | 1 | 1 | 1 | 2 |
| Dietician | 1 | 1 | 1 | 2 |
| Medical Officer | 14 | 27 | 27 | 52 |
| Dermatologist | 1 | 1 | 1 | 2 |
| Doctor Anaesthetist | 2 | 3 | 3 | 6 |
| ENT Specialist | 1 | 1 | 1 | 2 |
| Internal Medicine Physician | 1 | 2 | 3 | 5 |
| Neurosurgeon | 1 | 1 | 1 | 2 |
| Obstetrician & Gynaecologist | 2 | 4 | 4 | 8 |
| Ophthalmologist | 1 | 2 | 2 | 3 |
| Paediatrician | 2 | 3 | 3 | 5 |
| Trauma &Orthopaedic Specialist | 1 | 1 | 1 | 2 |
| Urologist | 1 | 1 | 1 | 2 |
| General Surgeon | 2 | 3 | 3 | 5 |
| Paediatric Surgeon | 1 | 2 | 2 | 3 |
| Midwife | 21 | 40 | 48 | 92 |
| Community Health Nurse/Field Technician | 4 | 7 | 8 | 16 |
| Enrolled Nurse | 80 | 123 | 125 | 142 |
| General Nurse | 121 | 142 | 185 | 355 |
| Critical Care Nurse | 4 | 7 | 8 | 15 |
| Emergency Nurse | 6 | 12 | 14 | 26 |
| ENT Nurse | 1 | 2 | 2 | 4 |
| Ophthalmic Nurses | 2 | 3 | 3 | 5 |
| Peri-Operative Nurses | 5 | 9 | 10 | 20 |
| Public Health Nurse | 3 | 6 | 6 | 11 |
| Nutritionist | 1 | 2 | 2 | 4 |
| Opticians/Optical Technician | 1 | 1 | 1 | 2 |
| Optometrist | 1 | 1 | 1 | 2 |
| Pharmacist | 6 | 12 | 18 | 35 |
| Pharmacy Technician | 3 | 6 | 12 | 23 |
| Certified Registered Anaesthetist | 9 | 12 | 12 | 18 |
| Physiotherapist | 2 | 3 | 4 | 7 |
| Physiotherapy Assistant | 3 | 5 | 5 | 10 |
| Mental Health Nurse | 3 | 6 | 6 | 12 |
| Psychiatrist | 1 | 1 | 1 | 2 |
| [Radiographer/X-ray Technician](../../../../D:/LIBRARY/WORKSHOPS%20AND%20CONFERENCES/WISIN%20Workshop/Dodowa%20Final%20Analysis/New%20-%20TH%20staffing%20Norm%20for%20sorted.xlsx#'Regional%20Hospital'!_ftn1) | 2 | 4 | 4 | 7 |
| Radiologist | 1 | 1 | 1 | 2 |
| Technical Assistant (X-Ray) | 1 | 2 | 2 | 4 |
| Artisans (Mechanical, Electricals, Carpentry, Mason) | 3 | 5 | 6 | 12 |
| Estates Officer | 1 | 1 | 1 | 2 |
| Health Services Administrator | 2 | 3 | 3 | 5 |
| Human Resource Manager | 1 | 2 | 1 | 3 |
| IT Managers and Officer | 2 | 3 | 4 | 7 |
| Procurement Officer | 1 | 1 | 1 | 2 |
| Supply Officer | 2 | 3 | 4 | 7 |
| Specialist Pharmacist - Cardiology | 1 | 1 | 1 | 2 |
| Specialist Pharmacist - Infectious Disease | 2 | 3 | 2 | 4 |
| Specialist Pharmacist - Oncology & Haematology | 1 | 2 | 1 | 3 |
| Specialist Pharmacist - Mental Health | 1 | 1 | 1 | 1 |
| Specialist Pharmacist - Paediatrics | 1 | 3 | 2 | 4 |
| Audiologist | 1 | 2 | 3 | 5 |
| Specialist Neonatologist | 2 | 3 | 3 | 5 |
| Neurosurgeon | 2 | 4 | 3 | 5 |
| Intensive Care Specialist | 5 | 8 | 8 | 16 |
| Occupational Therapist | 1 | 1 | 1 | 2 |
| Blood Donor Organiser | 1 | 1 | 1 | 2 |
| Phlebotomist | 4 | 10 | 15 | 23 |
| Blood bleeder | 1 | 3 | 5 | 7 |
| Catering Officer | 1 | 2 | 4 | 6 |
| Diet Cook | 1 | 3 | 4 | 7 |
| Hospitality Manager | 1 | 3 | 5 | 8 |
| Staff Cook | 1 | 2 | 2 | 4 |
| Clinical Psychologist | 1 | 1 | 2 | 3 |
| Technical Officer (Disease Control) | 1 | 1 | 2 | 2 |
| Driver | 6 | 8 | 8 | 12 |
| Executive Officer | 1 | 1 | 2 | 2 |
| Hospital Orderly | 10 | 15 | 23 | 35 |
| Labourer | 5 | 8 | 12 | 18 |
| Launderer | 4 | 6 | 9 | 14 |
| Mortuary Attendant | 1 | 2 | 2 | 3 |
| Private Secretary | 2 | 3 | 3 | 5 |
| Prosector | 1 | 1 | 1 | 2 |
| Receptionist/Telephonist | 1 | 2 | 3 | 5 |
| Seamstress | 1 | 2 | 4 | 5 |
| Security Guard | 1 | 1 | 2 | 3 |
| Stenographer | 1 | 2 | 3 | 5 |
| Sterilization Machine Operator | 3 | 5 | 6 | 9 |
| Transport Manager | 1 | 1 | 1 | 2 |
| Transport Officer | 1 | 1 | 2 | 3 |
| Burns and Plastic Nurse | 2 | 4 | 6 | 9 |
| Nephrology Nurse | 1 | 1 | 2 | 2 |
| Nephrology Paediatric Nurse | 1 | 1 | 2 | 2 |
| Public Health Nurse | 2 | 3 | 5 | 7 |
| Health Research Officer | 1 | 1 | 1 | 2 |
| Burns and Plastic and Reconstructive Surgeon | 1 | 1 | 1 | 2 |
| Neurosurgeon | 1 | 2 | 3 | 5 |
| Estate Manager | 1 | 1 | 1 | 2 |
| Auditor | 1 | 2 | 3 | 5 |
| Public Health Officer (Disease Control) | 1 | 2 | 3 | 5 |
| Public Health Officer (Health Promotion) | 1 | 2 | 3 | 5 |
| Technical Officer (Health Information) | 1 | 2 | 3 | 5 |

## 3.7 STAFFING NORMS FOR TEACHING HOSPITALS

Teaching Hospitals are nominally classified into Emerging (recently established or developing) and Established (well-developed) Teaching Hospitals.

Table S7: Staffing Norms for Teaching Hospitals

| **STAFF CATEGORY** | **Emerging TH Minimum** | **Emerging TH Maximum** | **Established TH Minimum** | **Established TH Maximum** |
| --- | --- | --- | --- | --- |
| Accountant | 8 | 15 | 20 | 26 |
| Accounts Officer | 24 | 43 | 57 | 76 |
| Finance Officer | 7 | 10 | 10 | 13 |
| Internal Auditor | 4 | 7 | 9 | 12 |
| Biomedical Scientist – Bacteriology | 14 | 25 | 33 | 44 |
| Biomedical Scientist – Biochemistry | 7 | 12 | 16 | 21 |
| Biomedical Scientist – Histopathology | 2 | 4 | 5 | 7 |
| Biomedical Scientist – Immunology | 8 | 14 | 18 | 24 |
| Biomedical Scientist – Serology | 5 | 9 | 12 | 15 |
| Biomedical Scientist - Haematology | 14 | 25 | 33 | 44 |
| Biomedical Scientist - Parasitology | 4 | 7 | 10 | 13 |
| Biostatistics Assistant | 12 | 23 | 36 | 48 |
| Biostatistics Officer | 4 | 7 | 11 | 15 |
| Technical Officer (Biostatistics/Health Information) | 6 | 12 | 18 | 24 |
| Clinical Engineering Manager | 2 | 5 | 5 | 8 |
| Clinical Engineering Technologist | 4 | 7 | 9 | 12 |
| Dental Prosthesis Technologist | 2 | 4 | 6 | 8 |
| Dental Prosthesis Technician | 2 | 3 | 4 | 6 |
| Dental Surgery Assistant | 2 | 4 | 6 | 8 |
| Maxillofacial surgeon | 2 | 4 | 5 | 7 |
| Specialist Community Oral Health /Dentist | 2 | 4 | 6 | 8 |
| Specialist Orthodontist | 1 | 1 | 2 | 2 |
| Specialist Restorative Dentistry | 2 | 3 | 4 | 6 |
| Dietician | 5 | 9 | 12 | 17 |
| Medical Officer (General Practitioner) | 38 | 60 | 65 | 85 |
| Cardiologist | 2 | 5 | 4 | 10 |
| Cardio-Thoracic Surgeon | 1 | 2 | 2 | 5 |
| Dermatologist | 1 | 3 | 2 | 5 |
| Doctor Anaesthetist | 11 | 20 | 27 | 36 |
| Emergency Medicine Physician | 9 | 17 | 22 | 30 |
| Endocrinologist | 1 | 3 | 2 | 5 |
| Family Physician | 3 | 5 | 7 | 9 |
| Gastroenterologist | 2 | 4 | 4 | 8 |
| Infectious Disease Specialist | 2 | 4 | 4 | 8 |
| Nephrologist | 1 | 2 | 3 | 4 |
| Neurologist | 1 | 2 | 2 | 4 |
| Obstetrician & Gynaecologist | 9 | 15 | 21 | 28 |
| Ophthalmologist | 3 | 5 | 7 | 9 |
| Paediatric Endocrinologist | 1 | 2 | 2 | 4 |
| Paediatric Nephrologist | 1 | 2 | 2 | 4 |
| Paediatric Neurologist | 1 | 2 | 2 | 4 |
| Paediatric Oncologist | 1 | 2 | 2 | 4 |
| Paediatrician (General) | 7 | 11 | 16 | 20 |
| Pathologist | 2 | 4 | 6 | 8 |
| Physician Specialist | 13 | 23 | 32 | 43 |
| Respiratory Physician | 1 | 3 | 2 | 5 |
| Rheumatologist | 1 | 1 | 2 | 2 |
| Specialist Haematology | 2 | 4 | 5 | 7 |
| Specialist Microbiology | 2 | 4 | 5 | 7 |
| Trauma & Orthopaedic Specialist | 2 | 4 | 6 | 8 |
| Urologist | 2 | 3 | 4 | 6 |
| General Surgeon | 5 | 8 | 11 | 14 |
| Paediatric Surgeon | 3 | 6 | 8 | 11 |
| Clinical Medical Physicist | 3 | 6 | 8 | 11 |
| Midwives | 98 | 175 | 234 | 310 |
| Community Health Nurse | 8 | 14 | 19 | 25 |
| General Nurses | 535 | 955 | 1279 | 1695 |
| Cardio-Thoracic Nurse | 36 | 65 | 87 | 116 |
| Critical Care Nurse | 76 | 136 | 183 | 242 |
| Emergency Nurse | 35 | 63 | 84 | 111 |
| ENT Nurse | 3 | 6 | 8 | 11 |
| ENT specialist | 2 | 4 | 5 | 7 |
| Oncology Nurse | 10 | 17 | 23 | 31 |
| Ophthalmic Nurse | 6 | 11 | 14 | 19 |
| Peri-Operative Nurse | 13 | 24 | 32 | 42 |
| Prosthesis Nurse (Occularist) | 1 | 1 | 1 | 2 |
| Nutritionist | 3 | 5 | 7 | 9 |
| Optical Technician | 1 | 1 | 1 | 2 |
| Optometrist | 2 | 3 | 4 | 6 |
| Clinical Pharmacist | 8 | 15 | 20 | 26 |
| Pharmacist | 32 | 57 | 76 | 101 |
| Pharmacy Specialist - Drug Information | 4 | 7 | 10 | 13 |
| Pharmacy Specialist- Manufacturing | 3 | 6 | 7 | 10 |
| Pharmacy Specialist –Radio | 3 | 5 | 7 | 9 |
| Pharmacy Technician | 21 | 38 | 51 | 67 |
| Physician Assistant (Anaesthesia) | 5 | 8 | 11 | 14 |
| Physiotherapist | 12 | 22 | 29 | 39 |
| Physiotherapy Assistant | 5 | 9 | 12 | 15 |
| Mental Health Nurse | 6 | 11 | 14 | 19 |
| Psychiatrist | 2 | 3 | 4 | 6 |
| Radiation Oncologist | 4 | 7 | 9 | 12 |
| Radiographers | 6 | 12 | 12 | 25 |
| X-ray Technician | 7 | 13 | 17 | 23 |
| Radiologist | 3 | 5 | 7 | 9 |
| Radiotherapist | 2 | 4 | 6 | 8 |
| Artisan (Mechanical, Plumbing, Electricals, Carpentry, Masonry) | 14 | 25 | 33 | 44 |
| Health Services Administrator | 7 | 13 | 17 | 23 |
| Human Resource Manager | 4 | 7 | 10 | 23 |
| IT Manager | 5 | 9 | 12 | 17 |
| Procurement Manager | 3 | 6 | 8 | 11 |
| Supply Officer | 12 | 22 | 30 | 40 |
| Audiologist | 1 | 1 | 2 | 4 |
| Specialist Pharmacist - Cardiology | 3 | 5 | 3 | 6 |
| Specialist Pharmacist - Infectious Disease | 4 | 7 | 6 | 11 |
| Specialist Pharmacist – Oncology & Hematology | 1 | 2 | 2 | 4 |
| Specialist Pharmacist – Mental Health | 3 | 5 | 4 | 6 |
| Specialist Pharmacist – Paediatrics | 6 | 9 | 10 | 15 |
| Occupational Therapist | 1 | 2 | 2 | 3 |
| Occupational Therapy Assistant | 1 | 3 | 1 | 3 |
| Blood Donor Organizer | 3 | 5 | 5 | 9 |
| Phlebotomist/Blood Bleeder | 12 | 23 | 23 | 45 |
| Catering Officer | 3 | 5 | 4 | 6 |
| Diet Cook | 3 | 4 | 3 | 5 |
| Staff Cook | 4 | 6 | 5 | 8 |
| Cook | 9 | 15 | 12 | 18 |
| Hospitality Manager (Catering) | 2 | 3 | 3 | 4 |
| Hospitality Manager (Institutional) | 2 | 2 | 3 | 6 |
| Health Planner | 1 | 2 | 2 | 3 |
| Clinical Psychologist | 4 | 7 | 6 | 10 |
| Health Research Officer | 3 | 6 | 5 | 8 |
| Public Health Officer (Disease Control) | 1 | 2 | 2 | 3 |
| Technical Officer (Disease Control) | 2 | 3 | 2 | 4 |
| Driver | 6 | 12 | 10 | 18 |
| Launderer | 11 | 22 | 21 | 40 |
| Hospital Orderly | 22 | 40 | 45 | 65 |
| Mortuary Attendant | 6 | 12 | 9 | 18 |
| Private Secretary | 8 | 16 | 13 | 25 |
| Prosector | 2 | 3 | 3 | 5 |
| Receptionist/Telephonist | 5 | 10 | 6 | 12 |
| Seamstress | 3 | 5 | 4 | 7 |
| Security Guard | 13 | 25 | 31 | 59 |
| Sterilization Machine Operator | 4 | 8 | 6 | 12 |
| Transport Manager | 2 | 3 | 2 | 3 |
| Burns and Plastics Nurse | 4 | 8 | 16 | 30 |
| Nephrology Nurse | 13 | 25 | 20 | 38 |
| Nephrology Paediatric Nurse | 5 | 9 | 8 | 15 |
| Public Health Nurse | 11 | 15 | 12 | 22 |
| Neurosurgeon | 2 | 4 | 3 | 5 |
| Intensive Care Specialist | 5 | 8 | 8 | 16 |
| Specialist Neonatologist | 2 | 3 | 3 | 5 |
| Dispensing Assistant | 19 | 28 | 25 | 35 |
| Respiratory Therapist | 3 | 5 | 4 | 7 |
| Executive Officer | 4 | 8 | 7 | 16 |
| Speech Therapist | 2 | 3 | 2 | 3 |

## 3.8 STAFFING NORMS FOR DISTRICT/MUNICIPAL/METROPOLITAN HEALTH DIRECTORATES

Metropolitan, Municipal and District Health Directorates are nominally classified based on the prevailing politico-administrative classification. Staffing norms for MMDHDs are provided in Table S8.

Table S8: Staffing Norms for District/Municipal/Metropolitan Health Directorates

| **Category** | **District Health**  **Directorate** | | **Municipal**  **Health Directorate** | | **Metropolitan**  **Health Directorate** | |
| --- | --- | --- | --- | --- | --- | --- |
|  |  |  |  |  |  |  |
|  | **Min** | **Max** | **Min** | **Max** | **Min** | **Max** |
| Technical Officer (Disease Control) | 2 | 3 | 2 | 3 | 2 | 4 |
| Public Health Officer (Disease Control) | 1 | 1 | 1 | 1 | 2 | 4 |
| Technical Officer (Health Information) | 2 | 2 | 2 | 3 | 2 | 3 |
| Public Health Officer (Health Information) | 1 | 1 | 1 | 1 | 1 | 2 |
| Public Health Officer (Health Promotion) | 1 | 1 | 1 | 2 | 2 | 2 |
| Technical Officer (Health Promotion) | 1 | 1 | 1 | 2 | 2 | 1 |
| Public Health Officer (Nutrition) | 1 | 1 | 1 | 2 | 1 | 2 |
| Technical Officer (Nutrition) | 2 | 3 | 2 | 3 | 1 | 2 |
| Accountant | 1 | 1 | 1 | 1 | 1 | 2 |
| Finance Officer | 2 | 2 | 2 | 3 | 2 | 3 |
| Auditor | 1 | 1 | 1 | 1 | 1 | 1 |
| Driver | 1 | 2 | 2 | 3 | 2 | 3 |
| Estate Officer | 1 | 1 | 1 | 2 | 1 | 2 |
| Labourer | 1 | 2 | 1 | 2 | 1 | 2 |
| Public Health Nurse | 1 | 2 | 1 | 2 | 2 | 3 |
| Health Research Officer | 1 | 1 | 1 | 1 | 1 | 1 |
| Executive Officer (HR Officer) | 1 | 1 | 1 | 1 | 1 | 2 |
| Human Resource Manager | 1 | 1 | 1 | 1 | 1 | 1 |
| Administrative Manager/Health Services Administrator | 1 | 1 | 1 | 1 | 1 | 2 |
| Procurement Manager | 1 | 1 | 1 | 1 | 1 | 1 |
| Supply Officer | 1 | 1 | 1 | 2 | 1 | 2 |
| Pharmacist | 1 | 1 | 1 | 1 | 1 | 1 |

**3.9 STAFFING NORMS FOR REGIONAL HEALTH DIRECTORATES**

Regional Health Directorates are not classified, thus generic staffing norms are provided in Table S9. However, it is envisaged that regional health directorates that serve larger populations may require additional staff as compared to those that serve a relatively fewer population but within the limits provided herein.

Table S9: Staffing Norms for Regional Health Directorates

| **CATEGORY** | **MIN** | **MAX** | **REMARKS** |
| --- | --- | --- | --- |
| Clinical Engineering Manager | 1 | 2 |  |
| Clinical Engineering Technologist | 2 | 3 |  |
| Artisans (Plumber, Carpenter, Electrician) | 3 | 6 |  |
| Estate Manager | 1 | 2 |  |
| Estate Officer | 1 | 2 |  |
| Accountant | 4 | 6 |  |
| Auditor | 1 | 2 |  |
| Finance Officer | 3 | 5 |  |
| Administrative Manager | 2 | 3 |  |
| Executive Officer | 5 | 8 | Including HR Officers |
| Human Resource Manager | 3 | 6 |  |
| IT Manager | 1 | 2 |  |
| Hospital Orderly | 2 | 3 |  |
| Private Secretary | 1 | 2 |  |
| Procurement Manager | 1 | 2 |  |
| Security Guard | 3 | 5 | For RHD only |
| Biostatistics Officer/Public Health Officer (Health Information)/Statistician | 2 | 3 |  |
| Technical Officer (Health Information) | 5 | 8 |  |
| Public Health Officer (Health Promotion)/Health Promotion Manager | 1 | 2 |  |
| Technical Officer (Health Promotion) | 2 | 3 |  |
| Nutrition Officer/Public Health Officer (Nutrition) | 1 | 2 |  |
| Technical Officer (Nutrition) | 2 | 3 |  |
| Public Health Officer (Disease Control) | 1 | 2 |  |
| Technical Officer (Disease Control) | 1 | 2 |  |
| Pharmacist | 1 | 2 |  |
| Public Health Nurse | 2 | 3 |  |
| Registered General Nurse (Not below PNO) | 2 | 4 |  |
| Registered Midwife (Not below PNO) | 1 | 2 |  |
| Health Planner | 1 | 2 |  |
| Health Research Officer | 1 | 2 |  |
| Storekeeper | 3 | 5 |  |
| Supply Manager | 1 | 2 |  |
| Supply Officer | 1 | 2 |  |
| Driver | 10 | 15 |  |
| Mechanical Engineer | 1 | 2 |  |
| Mechanical Engineer Technologist | 1 | 2 |  |
| Transport Manager | 1 | 2 |  |
| Transport Officer | 1 | 2 |  |
| **REGIONAL MEDICAL STORES** | | | |
| Pharmacist | 3 | 4 | Where production is done, the maximum is 6 |
| Pharmacy Technician | 2 | 3 | Where production is done, the maximum is 7 |
| Supply Manager | 1 | 2 |  |
| Supply Officer | 3 | 4 |  |
| Hospital Orderly | 2 | 3 |  |
| Stenographer Secretary | 1 | 1 |  |
| Security Guard | 2 | 3 |  |
| Accountant | 1 | 1 |  |
| Finance Officer | 1 | 1 |  |
| **REGIONAL MECHANICAL WORKSHOP** | | | |
| Mechanical Engineering Manager | 1 | 2 |  |
| Mechanical Engineering Technologist | 3 | 5 |  |
| Artisan (Electrical) | 1 | 2 |  |
| Artisan (Mechanical) | 4 | 7 |  |
| Accountant or Finance Officer | 1 | 1 |  |

**3.10 STAFFING NORMS FOR HEALTH TRAINING INSTITUTIONS**

Health Training Institutions are not categorised; hence a generic staffing norm is provided in Table S10 for selected categories of staff. However, when new programmes are to be introduced in an existing HTI, the formula below should be used to adjust the aggregate number of tutors needed in the institution (but within the limits of the norm provided herein).

**Table S10: Staffing Norms for Health Training Institutions**

| ***Number of Tutors = 1.44 + (1.72 X No. of Programmes) + (1.97 X Number of Classrooms)*** |
| --- |

| **CATEGORY** | **MINIMUM** | **MAXIMUM** | **REMARKS** |
| --- | --- | --- | --- |
| Accountant | 1 | 2 |  |
| Administrative Manager | 1 | 2 |  |
| Catering Officer | 2 | 3 |  |
| Driver | 2 | 4 |  |
| Finance Officer | 3 | 5 |  |
| Hostel Warden | 1 | 3 |  |
| IT Manager | 1 | 2 |  |
| Labourer | 2 | 5 |  |
| Supply Officer | 1 | 1 |  |
| Librarian | 3 | 5 |  |
| Security Guard/Watchman | 7 | 12 |  |
| Executive Officer | 2 | 4 |  |
| Health Tutors | 13 | 51 | See formula above |

**Appendix 1: How to make adjustments in the staffing requirements using the norms**

Following statistical test runs, the following guide may be used to make projections for staffing requirements in a facility based on this staffing norm.

15% change in workload leads to about 23. 5% change in Staffing requirement

10% change in workload leads to 14. 3% change in staffing requirement

5% change in workload leads to 6.3% change in staffing requirement

Where the workload change far exceeds 15%, it is advisable to check if the workload category has changed or not

Any percentage change in workload within the points of 5%, 10% and 15% should assume the status quo of the lower level. For example, 8% increase in workload will assume the staffing requirement at 5%.

1. *In accordance with the transport policy one driver per functional car* [↑](#footnote-ref-2)
2. . *The use of OPD and admissions as co-indicators reduces the chance of unnecessary shifting due to unusual happenings e.g. a one-off outbreak of disease* [↑](#footnote-ref-3)
3. *. Admission as a percentage of OPD is around 5%-11% (9.5% on the average) except a few outliers where up to 17% of OPD cases are admitted.* [↑](#footnote-ref-4)
